# Synthesis of Ultrathin Biotite Nanosheets as an Intelligent Theranostic Platform for Combination Cancer Therapy

**DOI:** 10.1002/advs.201901211

**Published:** 2019-08-20

**Authors:** Xiaoyuan Ji, Yong Kang, Jiang Ouyang, Yunhan Chen, Dolev Artzi, Xiaobin Zeng, Yuling Xiao, Chan Feng, Baowen Qi, Na Yoon Kim, Phei Er Saw, Na Kong, Omid C. Farokhzad, Wei Tao

**Affiliations:** ^1^ Center Lab of Longhua Branch, and Department of Infectious Disease Shenzhen People's Hospital 2nd Clinical Medical College of Jinan University Shenzhen 518120 Guangdong Province China; ^2^ Integrated Chinese and Western Medicine Postdoctoral Research Station Jinan University Guangzhou 510632 China; ^3^ Center for Nanomedicine Brigham and Women's Hospital Harvard Medical School Boston MA 02115 USA; ^4^ State Key Laboratory of Biochemical Engineering Institute of Process Engineering Chinese Academy of Sciences Beijing 100190 China; ^5^ Guangdong Provincial Key Laboratory of Regional Immunity and Diseases School of Medicine Shenzhen University Shenzhen 518061 China

**Keywords:** 2D nanosheets, biotite, combination cancer therapy, reactive oxygen species

## Abstract

Biotite, also called black mica (BM), is a group of sheet silicate minerals with great potential in various fields. However, synthesis of high‐quality BM nanosheets (NSs) remains a huge challenge. Here, an exfoliation approach is provided that combines calcination, *n*‐butyllithium exchange and intercalation, and liquid exfoliating processes for the high‐yield synthesis of ultrathin BM NSs. Due to the presence of MgO, Fe_2_O_3_, and FeO in these NSs, PEGylated BM can be engineered as an intelligent theranostic platform with the following unique features: i) Fe^3+^ can damage the tumor microenvironment (TME) through glutathione consumption and O_2_ production; ii) Generated O_2_ can be further catalyzed by MgO with oxygen vacancy to generate ·O_2_^−^; iii) The Fe^2+^‐catalyzed Fenton reaction can produce ·OH by disproportionation reactions of H_2_O_2_ in the TME; iv) Reactions in (i) and (iii) circularly regenerate Fe^2+^ and Fe^3+^ for continuous consumption of glutathione and H_2_O_2_ and constant production of ·OH and O_2_; v) The NSs can be triggered by a 650 nm laser to generate ·O_2_^−^ from O_2_ as well as by an 808 nm laser to generate local hyperthermia; and vi) The fluorescent, photoacoustic, and photothermal imaging capabilities of the engineered NSs allow for multimodal imaging‐guided breast cancer treatment.

Reactive oxygen species (ROS), including hydroxyl radicals (·OH), singlet oxygen (^1^O_2_), and superoxide ion (·O_2_^−^), have been regarded as effective therapeutic agents for cancer due to their ability to cause irreversible permanent damage to the cells and eventually induce cell apoptosis.^1^ One of the most extensively explored ROS‐mediated cancer therapies, photodynamic therapy (PDT), has numerous superiorities (e.g., high selectivity, minimal invasiveness, and low side effects) over other conventional therapies. Nevertheless, the clinical application of PDT is largely limited by the existing problems of traditional organic photosensitizers, including poor stability, low solubility, low loading capacity, and uncontrollable release from the carriers.^2^ Although some semiconductors are reported to have better biocompatibility, photostability, and light responsiveness than conventional organic photosensitizers, they still suffer from low ROS generation rate in PDT due to the limitation of the hypoxic tumor microenvironment (TME).^3^

On account of the relatively high content of H_2_O_2_ in the TME, Fe^3+^/Fe^2+^‐mediated chemodynamic therapy (CDT) has shown a promising antitumor activity through Fenton reactions.^4^ The Fenton reaction generates the most toxic ROS (·OH) via disproportionation reactions of H_2_O_2_ in a tumor‐specific microenvironment, which largely avoids damage to normal tissues. It also produces O_2_ in excess of H_2_O_2_, providing substrate supplement for PDT to some extent.[qv: 4a] Hence, constructing a multiple ROS‐mediated platform integrating the Fenton reaction into PDT is a hopeful strategy for synergistic antitumor effects.

2D nanomaterials with ultrathin structure and fascinating physiochemical properties have now attracted considerable attention.^5^ Mica, one of the sheet‐like natural layered clays, has attracted extensive attention because of its impressive physiochemical properties.^6^ Biotite, also named black mica (hereinafter BM; approximate chemical formula: K(Mg,Fe)_3_AlSi_3_O_10_(F,OH)_2_), is one of the most important structures of mica, which not only consists of an octahedral layer of aluminum oxide (Al_2_O_3_) sandwiched between two identical tetrahedral layers of silicon oxide (SiO_2_), but also doped with other metal oxides, including magnesium oxide (MgO), ferric oxide (Fe_2_O_3_), and ferrous oxide (FeO). The unique composition of BM endows it great potential for designing a multiple ROS‐mediated cancer therapy that combines PDT and CDT. Specifically, the BM‐based platform has the potential to perform the following functions: Fe^3+^‐catalyzed damage of the TME (i.e., consuming glutathione (GSH) and producing O_2_), Fe^2+^‐catalyzed Fenton reaction to produce ·OH, and MgO‐catalyzed generation of ·O_2_^−^ from O_2_. Moreover, doping with new metal elements (Mg and Fe) can further change its electronic properties, such as a more tunable bandgap, light absorption, and transformation, which make it uniquely promising for PDT, as well as additional property for photothermal therapy (PTT). Since BM is commonly used as a ayurvedic drug formulation for the treatment of diseases, such as hepatitis (hepatoprotective),^7^ indicating the potential biocompatibility of BM‐based materials. However, unlike other layered 2D materials (i.e., graphene, black phosphorus, transition metal sulfide, and so on) which have weak van der Waals forces between the layers and strong in‐plane bonds,^8^ BM has extremely strong interlayer forces attributed to its bad swelling and cation exchange capacity, which makes it challenging to split into sheets consisting of a single or a few layers.[qv: 6a,9]

Herein, for the first time, a novel strategy to fabricate ultrathin BM NSs was developed through combining grinding, calcination, *n*‐butyllithium exchange and intercalation, and liquid exfoliating processes (**Scheme** 1). To further improve the dispersibility and biocompatibility of BM NSs, positively charged amine functionalized polyethylene glycol (PEG‐NH_2_) was absorbed on the negatively charged surface of BM NSs by electrostatic attraction. The prepared BM‐PEG NSs had a bandgap of 1.45 eV and were light‐responsive. When exposed to light (650 nm), BM‐PEG NSs generated electrons and hole pairs, which made them suitable for type I PDT that produces ·O_2_^−^ from O_2_. The MgO component of BM‐PEG NSs with oxygen vacancy catalyzed O_2_ transfer to ·O_2_^−^, which further increased the concentration of ·O_2_^−^. The redox pairs (Fe^2+^/Fe^3+^) could produce ·OH through the Fenton reaction by disproportionation reactions of H_2_O_2_. Upon irradiation by 650 nm light, BM‐PEG NSs could trigger more production of ·OH, which could enhance the therapeutic effect of CDT. Meanwhile, the BM‐PEG NSs were able to modulate TME by depleting GSH and generating O_2_ via the high oxidability of Fe^3+^. This decreased the antioxidant capability and hypoxia of tumors, which further allowed BM‐PEG NSs to perform photoenhanced CDT and improved PDT. BM‐PEG NSs also exhibited high photothermal conversion efficacy under the irradiation of an 808 nm laser, achieving prominent synergistic and photoenhanced CDT/PDT/PTT in vitro and in vivo. Moreover, the functionalized BM‐PEG NSs showed excellent photothermal, photoacoustic (PA) and fluorescence signals, demonstrating that BM‐PEG NSs could act as photothermal, PA, and fluorescent imaging agents. Therefore, this study not only provided a novel approach for the synthesis of ultrathin BM NSs, but also developed an intelligent BM‐based theranostic platform for multimodal imaging‐guided cancer treatment.

**Scheme 1:**
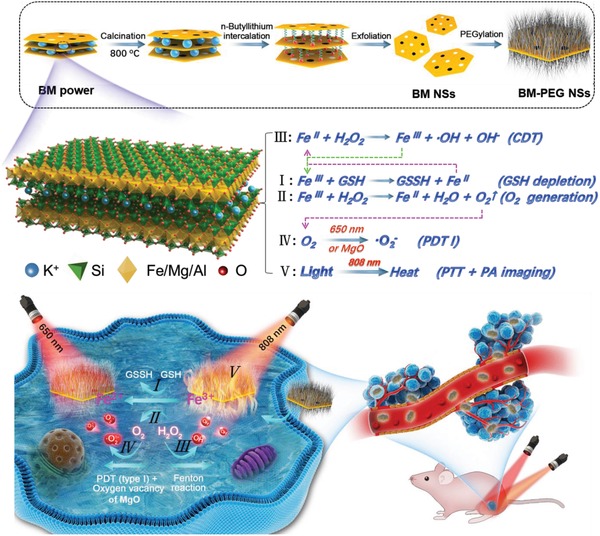
Schematic illustration of the preparation process and therapeutic mechanism of BM‐PEG NSs.

The successful synthesis of 2D BM NSs in this study was achieved by the combination of grinding, calcination, *n*‐butyllithium exchange and intercalation, and liquid exfoliating processes (Scheme 1). Due to the large and nonuniform particle sizes of the purchased BM powder, the BM powder was wet ground in *N*‐methyl pyrrolidone (NMP) until the size was 500–800 nm (Figure S1, Supporting Information). Because of the extremely strong interlayer forces in BM, the key to the successful exfoliation of BM NSs is to decrease the cohesion energy and open up the crystallite of BM.^10^ As K^+^ is firmly locked in the interlayers of BM to counterweigh the charge due to the severe charge deficiency of BM layers, calcination at 800 °C was used to decrease the attraction force between the layers and K^+^.^11^ In the past decade, lots of previous studies have tried to weaken the interlayer attraction force and increase the interlayer spacing of mica.^12^ For example, due to the small size and positive charge of Li^+^, Li^+^ was used to extract K^+^ from the interlayers.^13^ Then, long‐chain positive ion polymers, such as the octadecyl trimethyl ammonium ion or hexadecyl trimethyl ammonium bromide, were employed to intercalate into the interlayers of mica to further expand the interlayer spacing.[qv: 6a,10,11] In this study, *n*‐butyllithium with a Li^+^ head and relatively long‐chain tail was chosen to achieve K^+^ extraction and interlayer spacing expansion in a single step. The combination of calcination treatment and *n*‐butyllithium intercalation largely decreased the attraction force and expanded the interlayers spacing of BM. Ultrasound‐facilitated exfoliation was then applied to completely break down the reduced interlayer attraction force to get a single or a few layers of BM NSs (the atomic structure of single layer BM NS was shown in Figure S2 in the Supporting Information). As shown in Figures S3 and S4 in the Supporting Information, after liquid exfoliation, the average size and thickness of BM NSs were ≈120 and ≈3 nm, respectively. Next, positively charged PEG‐NH_2_ was utilized to modify the surface of BM NSs by electrostatic attraction to further improve their dispersibility and physiological stability. The average size and thickness of BM‐PEG NSs were changed to ≈105 and ≈6 nm, respectively, which also demonstrated the success of PEGylation (**Figure** 1a,b). After PEGylation, the BM‐PEG NSs exhibited great dispersity and stability in phosphate buffer saline (PBS) and in medium solution (Figure S5, Supporting Information). However, without well‐prepared process, the produced larger (without complete grinding and liquid exfoliating) or thicker (without complete *n*‐butyllithium exchange and intercalation) BM‐PEG NSs showed poor dispersity in PBS and in medium solution even after PEGylation, reiterating the necessary and superiority of our synthetic methodology. Figure 1c shows the X‐ray diffractometry (XRD) spectrum of the crystal structure of fabricated BM NSs, which is consistent with JCPDS No. 00‐042‐1437. To corroborate the successful PEGylation of BM NSs, Fourier‐transform infrared (FT‐IR) and X‐ray photoelectron spectroscopy (XPS) spectra were applied to analyze the composition of BM NSs before and after PEGylation (Figure 1d,e). In the XPS analysis, two new peaks of C and N appeared in the XPS spectra after PEGylation, which provides clear evidence for successful PEGylation. In addition, as shown in Figure 1e, the absorption bands at about 1250 and 2900 cm^−1^, which were contributed by stretching vibrations of C=O and CH of PEG‐NH_2_ respectively, further indicating the successful surface coating by PEG‐NH_2_. Moreover, the elements of BM, such as Al, Fe, Mg, and Si, and the elements of PEG‐NH_2_, such as C, N, and O, were all shown in the energy dispersive spectrometer (EDS) mapping of PEGylated BM NSs (Figure 1h), which indicates the successful surface modification of BM NSs. In addition, the contents of Fe and Mg in BM NSs were calculated to be about 9% and 14% based on XPS and EDS analyses, respectively. Although the composition may slightly vary with the producing areas of BM, the preparation method combining grinding, calcination, *n*‐butyllithium exchange and intercalation, and liquid exfoliating processes could be applicable to the exfoliation of all kinds of BM, due to the fact that all BMs have the same structure, K^+^, locked in the interlayers of sandwiched oxides.

**Figure 1:**
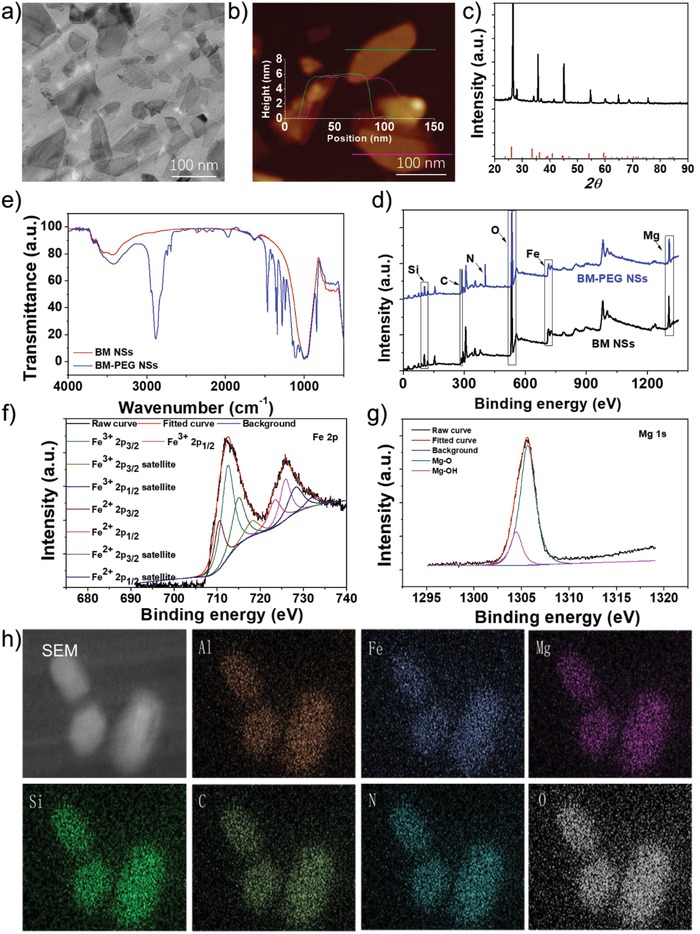
Characterization of ultrathin 2D BM‐PEG NSs. a) TEM images, b) AFM images, c) XRD spectra, d) FTIR spectra, e) XPS spectra, f) HRXPS spectra of Fe, g) HRXPS spectra of Mg, and h) EDS images of BM‐PEG NSs.

The ability of BM‐PEG NSs to induce NIR‐mediated hyperthermia was evaluated by testing their light absorbance, photothermal conversion efficiency, as well as photothermal stability. The light absorbance of BM NSs and BM‐PEG NSs ranging from UV to NIR shows that both BM NSs and BM‐PEG NSs have a strong and broad absorption of light (**Figure** 2a; Figures S6 and S7, Supporting Information). To evaluate the photothermal conversion ability of BM‐PEG NSs, the aqueous solutions of BM‐PEG NSs at different concentrations, ranging from 50 to 200 µg mL^−1^, were prepared and exposed to an 808 nm laser at power densities of 1, 1.5, and 2 W cm^−2^ for 5 min. The photothermal heating curves (Figure 2b; Figure S8, Supporting Information) exhibit a rapid and large temperature increase of the BM‐PEG NS solution. Concentration‐dependent and laser power‐dependent photothermal effects of BM‐PEG NSs were also observed. This was especially the case when BM‐PEG NS solution (200 µg mL^−1^) was irradiated under an 808 nm light at 2 W cm^−2^ for 5 min, with the highest temperature increase (Δ*T*_max_) of 36 °C. As a reference, when pure water was tested under the same conditions, Δ*T*_max_ of pure water was only 4 °C. However, without well‐prepared process, the BM‐PEG NSs (with an average size of ≈270 nm or an average thickness of ≈35 nm) have much lower exposed specific area to absorb NIR, which led to the Δ*T*_max_ reaching only 29 and 26 °C after 5 min of NIR laser irradiation at 2 W cm^−2^ (Figure S9, Supporting Information). The enhanced Δ*T*_max_ further confirmed the superiority of our strategy for the preparation of ultrathin BM‐PEG NSs. According to the photothermal heating and cooling curves (Figure S10, Supporting Information), the photothermal conversion efficiency (η) of BM‐PEG NSs was calculated to be 38.4% (Figure 2c). Although the η of BM‐PEG NSs was lower than that of recently developed new photothermal agents (PTAs), such as antimonene (45%),[qv: 8c,d] borophene (42%),^14^ and TaS_2_ NSs (39%),[qv: 8b] it was much higher than those of most PTAs, such as graphene oxide (25%),^15^ Cu_2−_ *_x_*Se nanoparticles (22%),^16^ and Au nanorods (21%).^17^ This data indicates that BM‐PEG NSs have great potential to act as effective PTAs. The photostability of BM‐PEG NSs was another important qualification for good PTAs. Figure S11 in the Supporting Information presents five cycles of heating and cooling temperature curves of BM‐PEG NSs solution under the irradiation of 808 nm light at 2 W cm^−2^. There was no obvious change in the photothermal effects of BM‐PEG NSs during five cycles, indicating their high photostability. In addition, the storage stability of BM‐PEG NSs was evaluated by continuously checking their vis–NIR absorbance and photothermal conversion. As shown in Figure S12 in the Supporting Information, after storing at room temperature for 30 days, there were no detectable changes of light absorbance and photothermal conversion of BM‐PEG NSs, which demonstrates their great storage stability.

**Figure 2:**
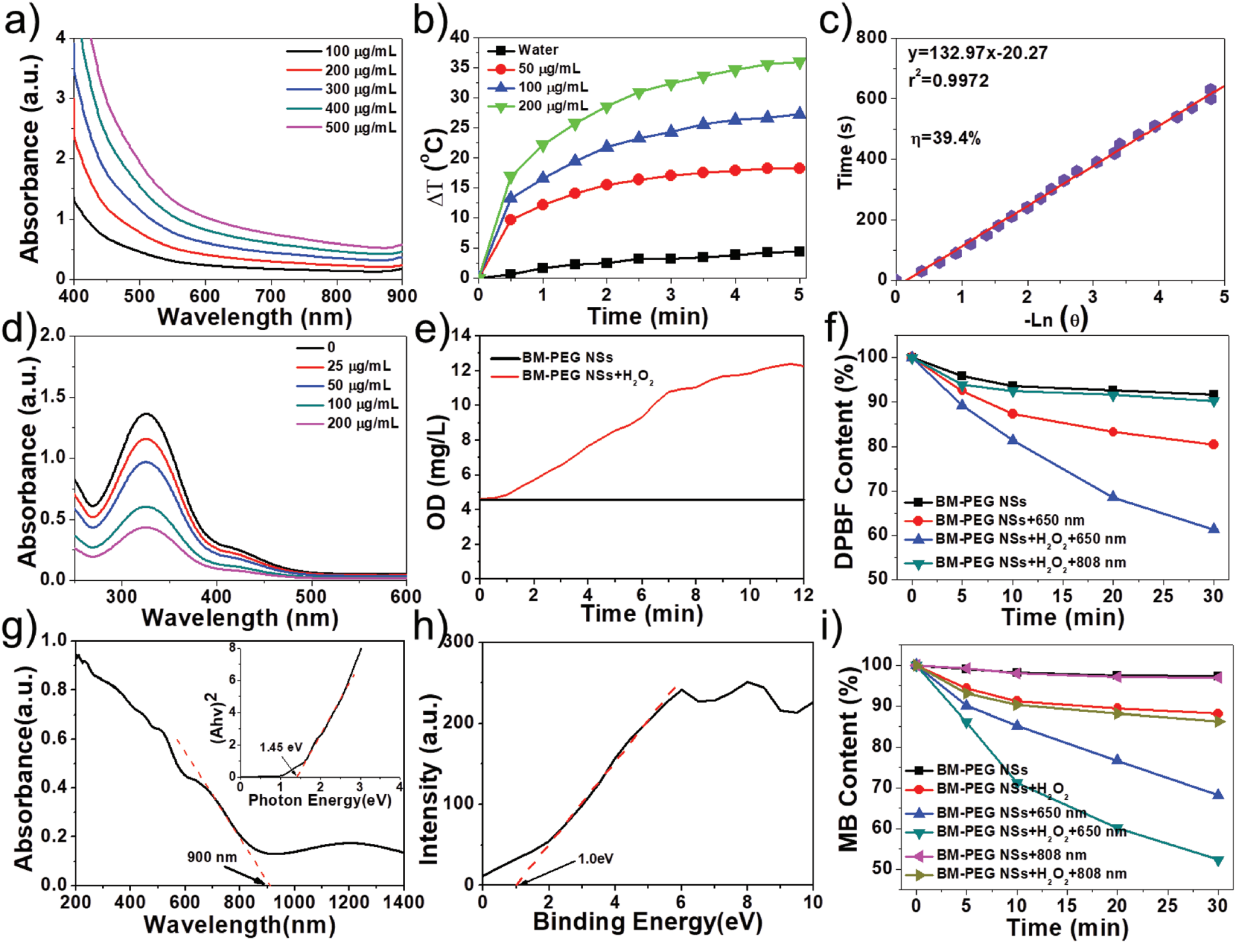
a) UV–vis–NIR absorbance spectra of BM‐PEG NSs. b) Temperature change curves of BM‐PEG NSs at different concentrations (0–200 µg mL^−1^) exposed to an 808 nm laser (2 W cm^−2^) for 5 min. c) Linear relationship between −ln θ and time. d) GSH depletion with different concentrations of BM‐PEG NSs (0–200 µg mL^−1^). e) O_2_ generation of BM‐PEG NSs with H_2_O_2_. f) Degradation of DPBF owing to ·O_2_^−^ generation under different conditions. g) UV–vis–IR diffuse reflection spectra and the corresponding bandgap of BM‐PEG NSs estimated from Kubelka–Munk equation. h) Conduction band of BM‐PEG NSs. i) Depletion of MB owing to ·OH production under different conditions.

In order to test the ability of BM‐PEG NSs to modulate the TME, such as their effects on the concentration of GSH and O_2_ through Fe^3+^ and Fe^2+^ mediated redox reactions, the key components of BM‐PEG NSs were analyzed. Figure 1e,f exhibits the survey and high‐resolution XPS spectra of BM‐PEG NSs, respectively. The Fe 2p spectra consist of eight peaks at 712.55, 725.85, 718.30, 728.35, 710.55, 723.55, 715.05, and 732.05 eV. The peaks at 712.55, 725.85, 718.30, and 728.35 eV were assigned to Fe^3+^ 2p_3/2_, Fe^3+^ 2p_1/2_, Fe^3+^ 2p_3/2_ satellite, and Fe^3+^ 2p_1/2_ satellite, respectively. The peaks at 710.55, 723.55, 715.05, and 732.05 eV were ascribed to Fe^2+^ 2p_3/2_, Fe^2+^ 2p_1/2_, Fe^2+^ 2p_3/2_ satellite, and Fe^2+^ 2p_1/2_ satellite respectively. As shown in Figure 1f, there was abundant Fe^3+^ in BM‐PEG NSs, which could be reduced by GSH or H_2_O_2_ to decrease the antioxidant capability and hypoxia of tumors. The GSH consumption of BM‐PEG NSs is shown in Figure 2d, in which a rapid decrease of the GSH concentration dependent on the concentration of BM‐PEG NSs was observed. As reported in previous studies, the depletion of GSH would notably decrease the antioxidant capability of the tumor. Due to the Fe^3+^ component of BM‐PEG NSs, which could generate O_2_ by reacting with H_2_O_2_, mild O_2_ generation was observed when H_2_O_2_ was added to the BM‐PEG NS solution (Figure 2e). This data confirms that BM‐PEG NSs could diminish the tumor's hypoxia. The GSH depletion and O_2_ generation demonstrated that BM‐PEG NSs may regulate the TME in favor of ROS mediated therapies.

The potential of BM‐PEG NSs to serve as photosensitizers in PDT was evaluated via the ROS sensor agent 1,3‐diphenylisobenzofuran (DPBF), shown in Figure 2f and Figure S13 in the Supporting Information. Figure 2f shows that there was a slight decrease in DPBF when BM‐PEG NSs were used alone, which was mainly caused by the oxygen vacancy of nano‐MgO in BM‐PEG NSs that generated superoxide anion free radical, ·O_2_^−^, from O_2_. Compared to the BM‐PEG NSs, the absorbance of DPBF quickly decreased over time under 650 nm laser irradiation. The rapid degradation of DPBF was attributed to the sharp increase of ROS (·O_2_^−^) generated through photoexcited electron–hole pairs of BM‐PEG NSs, which is schematically illustrated in Figure S14 in the Supporting Information. In order to confirm the species of ROS, UV–vis–NIR diffuse reflectance spectra and XPS spectra were used to test the electronic band structure of BM‐PEG NSs. As shown in Figure 2g, BM‐PEG NSs had light absorptions throughout the whole visible and NIR region, with an absorption edge at around 900 nm. According to the Kubelka–Munk conversion, the bandgap (*E*_g_) of BM‐PEG NSs was calculated to be 1.45 eV (Figure 2g). Then, the valence band (VB) value of BM‐PEG NSs was determined by XPS spectra. As shown in Figure 2h, the VB value of BM‐PEG NSs was about 1.0 eV. The conduction band (CB) of BM‐PEG NSs was −0.45 eV, the difference between the *E*_g_ and VB. Because the reduction potential of O_2_/·O_2_^−^ was −0.16 eV, which was lower than the CB of BM‐PEG NSs (−0.45 eV), the transfer of photoexcited electrons from the CB to O_2_, which yields ·O_2_^−^, was thermodynamically favorable (Figure S14, Supporting Information). These results further demonstrate the great potential of BM‐PEG NSs as photosensitizers for efficient PDT. To mimic the TME, a certain amount of H_2_O_2_ was added to the above system. During the reaction, O_2_ was generated by the Fenton reaction catalyzed by Fe^3+^. The generated O_2_ replenished the substrate of PDT, which markedly enhanced ROS production. In the “BM‐PEG NSs + H_2_O_2_ + 808 nm light” group, the bandgap of BM‐PEG NSs was not small enough for the 808 nm laser to generate electron transition. Thus, the slightly enhanced ROS production ability was mainly caused by the increased O_2_ generation by the Fenton reaction, which allowed nano‐MgO's oxygen vacancy to generate ·O_2_^−^.

To test the ability of BM‐PEG NSs to produce ·OH through the Fenton reaction, methylene blue (MB) was selected as the ROS sensing agent. Figure 2i and Figure S15 in the Supporting Information show the degradation of MB in different conditions. A negligible change in the concentration of MB was observed when BM‐PEG NSs were used alone whereas a significant decrease of MB concentration was observed after adding H_2_O_2_, due to the plentiful ·OH generation by the Fenton reaction. Moreover, there was an apparent and sustained decrease of the MB concentration with irradiation at 650 nm. The primary reason for this could be that the photoexcited holes in the VB of BM‐PEG NSs played an important role in MB degradation. The influence of 808 nm light on the degradation of MB was also tested, as shown in Figure 2i. There was no obvious change between the groups with and without the irradiation at 808 nm. This phenomenon could be explained by the 808 nm light not having enough energy to support the separation of electron–hole pairs of BM‐PEG NSs. As mentioned before, the contents of Fe and Mg in BM may vary with the producing areas. However, the variation is very minimal and does not affect the structure of obtained BM NSs. Therefore, the properties of BM NSs, such as photomediated thermal conversion and ROS production, would be applicable to all BM NSs. Although the therapeutic effect of each BM might slightly vary, this does not diminish their potential for combination cancer therapy. Compared with the composition, the structure of BM NSs has a more pronounced effect on their performance. For example, BM‐PEG NSs with an average size of ≈270 nm or an average thickness of ≈35 nm have much less active sites on their surface, lessening their ability to cause the degradation of DPBF and MB via the production of ·O_2_^−^ and ·OH (Figure S16, Supporting Information).

After demonstrating the ability of BM‐PEG NSs to modulate TME and generate ROS, the in vitro TME‐modulating capacity and antitumor effect of BM‐PEG NSs were further tested. The cytotoxicity of BM‐PEG NSs was tested in A549, PC3, MCF7, and Hela cells by AlamarBlue assay. Without light exposure, BM‐PEG NSs at different concentrations did not induce obvious toxicity in four different human cancer cell lines (**Figure** 3a). The negligible cytotoxicity of BM‐PEG NSs not only demonstrates their potential biocompatibility after PEGylation, but also indicates no obvious active oxygen vacancy and Fenton reactions in the absence of light irradiation. The GSH‐consuming and O_2_‐generating capability of BM‐PEG NSs in MCF7 cells was tested by using the GSH assay kit and O_2_ probe [Ru(dpp)_3_]Cl_2_ (RDPP). As shown in Figure 3d, a decreased fluorescence signal was observed, which was mainly caused by the O_2_ evolution via the Fenton reaction. To test the depletion of GSH, MCF7 cells were treated with BM‐PEG NSs solutions at different concentrations (25, 50, 100, and 200 µg mL^−1^). After 24 h incubation, the GSH content decreased rapidly in a BM‐PEG NS concentration‐dependent manner (Figure 3b). To test the ROS generation of BM‐PEG NSs in MCF7, the ROS probe 2,7‐dichlorofluorescin diacetate (DCFH‐DA) was selected to analyze the intracellular ROS content. As exhibited in Figure 3c,e, the group treated with BM‐PEG NSs showed slightly stronger green fluorescence signals, as compared to the control and the 650 nm light‐alone group. ·O_2_^−^ and ·OH generation via the oxygen vacancy of MgO and the Fenton reaction in H_2_O_2_ abundant cancer cells explain these data. More interestingly, the cells treated with BM‐PEG NSs and 650 nm light showed much stronger green fluorescence emission, which implied more ·O_2_^−^ and ·OH generation by the photodynamic effect and the photoenhanced Fenton reaction. Next, the in vitro antitumor effect of BM‐PEG NSs was evaluated by testing cell viability after different treatments. Figure 3f and Figure S17 in the Supporting Information exhibited dose‐dependent cell death of BM‐PEG NSs‐treated cells exposed to an 808 or 650 nm light, showing the potential of BM‐PEG NSs to kill cancer cells via photothermal ablation or ROS alone. Moreover, nearly no cell survived when the cells were treated with BM‐PEG NSs and irradiated by both 808 and 650 nm lights, which highlighted the feasibility of using BM‐PEG NSs for effective photoenhanced CDT/PDT/PTT. The dead and live cells in the different treatment groups were stained with propidium iodide (PI) and calcein acetoxymethyl ester (calcein AM), respectively, to further demonstrate the effective antitumor effect of BM‐PEG NSs (Figure 3g).

**Figure 3:**
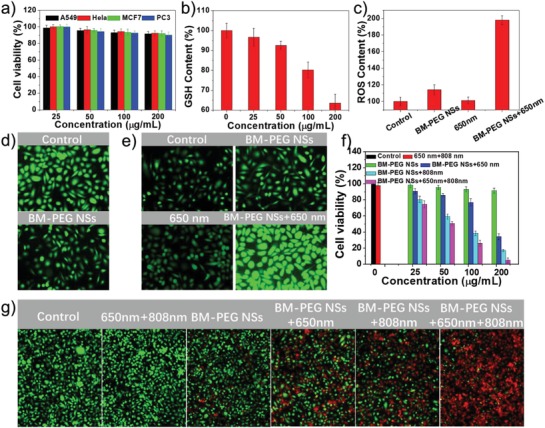
a) A549, Hela, MCF7, and PC3 cells viability under treatment with different concentrations of BM‐PEG NSs (25–200 µg mL^−1^) for 24 h. b) GSH content of MCF7 cells under treatment with different concentrations of BM‐PEG NSs (25–200 µg mL^−1^) for 24 h. c) ROS content of MCF7 cells under different treatments. d) Confocal laser scanning microscope (CLSM) images of O_2_ evolution of MCF7 cells without or with the treatment of BM‐PEG NSs. e) CLSM images of ROS generation under different treatments. Antitumor effect of BM‐PEG NS‐based varying treatments by f) AlamarBlue assay and g) CLSM images. For the 650 nm light treatment, the power density was 0.5 W cm^−2^, and the irradiation time was 10 min. For the 808 nm light treatment, the power density was 1.0 W cm^−2^, and the irradiation time was 10 min.

Based on the promising in vitro results, animal studies were executed to further explore the feasibility of BM‐PEG NSs as an intelligent in vivo theranostic platform. First, the MCF7 xenograft breast tumor model was established by planting MCF7 cells on the back of nude mice. The fluorescence imaging and biodistribution study of BM‐PEG NSs were performed via whole‐animal NIR imaging using BM‐PEG‐Cy7 NSs (Figure S18, Supporting Information) as an imaging agent and free Cy 7 as a reference. **Figure** 4a,b showed that BM‐PEG‐Cy7 NSs were largely accumulated at the tumor sites 24 h after injection, while there was nearly no accumulation of free Cy 7 at tumor sites. Additionally, pharmacokinetic studies were performed by detecting the fluorescence in blood at different time intervals (Figure S19, Supporting Information). Photoacoustic imaging with high sensitivity, spatial resolution, and deep penetration has become one of the most promising tools in cancer theranostics.^18^ Due the excellent photothermal performance of BM‐PEG NSs, the potential of BM‐PEG NSs as PA agent was assessed both in vitro and in vivo. As presented in Figure 4c,e, the concentration of BM‐PEG NSs and PA signal at 800 nm exhibited a strong linear relationship, which verified BM‐PEG NSs' great potential as PA agent for cancer imaging. Next, the BM‐PEG NSs were intravenously injected into MCF7 tumor‐bearing mice for PA imaging in vivo. At different time intervals, the PA signals of BM‐PEG NSs at the tumor site were recorded. Prominent PA signals were detected at the tumor sites 12 and 24 h postinjection of BM‐PEG NSs (Figure 4d). The quantitative analysis of PA signals is shown in Figure 4f, which validated BM‐PEG NSs' qualifications as a PA agent for imaging‐guided cancer theranostics.

**Figure 4:**
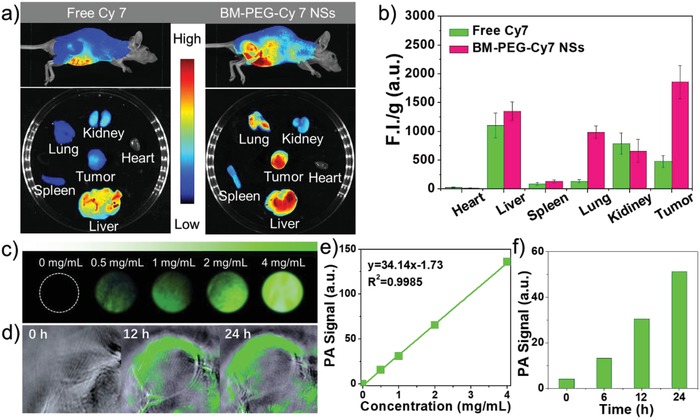
a) Fluorescence images of mice, major organs, and tumors 24 h after injection in vivo and ex vivo. b) Semiquantitative biodistribution of free Cy 7 or BM‐PEG‐Cy7 NSs in nude mice. c) PA images of different concentration of BM‐PEG NSs (0, 0.5, 1, 2, and 4 mg mL^−1^). d) PA images of the tumor site at different time intervals (0, 12, 24 h) postinjection. e) PA values of different concentration of BM‐PEG NSs (0, 0.5, 1, 2, and 4 mg mL^−1^). f) Semiquantitative analysis of PA values in (d).

After evaluating the feasibility of BM‐PEG NSs in fluorescence and PA dual‐modal imaging, in vivo studies on BM‐PEG NS‐based combinatorial cancer therapy were carried out. MCF7 tumor‐bearing mice were treated as described: Group 1: saline (control); Group 2: 650 nm + 808 nm lights (laser group); Group 3: BM‐PEG NSs (CDT group); Group 4: BM‐PEG NSs + 650 nm laser (photoenhanced CDT/PDT group); Group 5: BM‐PEG NSs + 808 nm laser (PTT group); and Group 6: BM‐PEG NSs + 650 nm + 808 nm (photoenhanced CDT/PDT/PTT group). 6 mg kg^−1^ of BM‐PEG NSs was intravenously injected into Groups 3, 4, 5, and 6. For the 650 nm light treatment, the power density was 0.5 W cm^−2^, and the irradiation time was 10 min. For the 808 nm light treatment, the power density was 1.0 W cm^−2^, and the irradiation time was also 10 min. Both light treatments were carried out at 24 h postinjection. For the mice treated with lasers, the change in temperature was detected by an IR thermal camera. An obvious increase (up to 15 °C) in temperature at the tumor site was shown in Group 5 while the temperature at tumor sites only increased by ≈3 °C in Groups 2 and 4 (**Figure** 5a,b). Such results demonstrated that BM‐PEG NSs were effective PTAs for locally heating the tumor with an 808 nm light. The tumor volumes in these six groups were recorded every 2 days, and the growth curves of tumors are shown in Figure 5c. The control group, BM‐PEG NSs‐only group, and laser‐only group showed no significant inhibition of tumor growth. An obvious inhibition of tumor‐growth was observed in both the PTT and photoenhanced CDT/PDT groups. More impressively, the tumors were completely ablated in the photoenhanced CDT/PDT/PTT group. The excised tumors from euthanized mice, as shown in Figure 5d, provided more direct evidence for the outstanding therapeutic effect of the photoenhanced CDT/PDT/PTT group. No notable side effects, including abnormal weight loss or perturbations in activity, drinking, and eating, were observed in the above treatment groups (Figure 5e).

**Figure 5:**
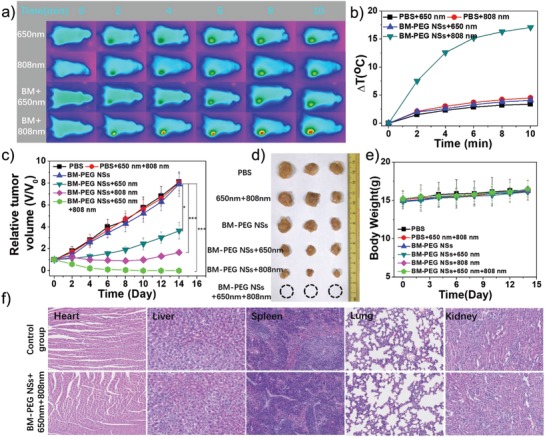
a) Infrared thermographic images and b) photothermal heating curves of breast tumor‐bearing nude mice under different treatments. c) Time‐dependent tumor volume of nude mice under different treatments. For the 650 nm light treatment, the power density was 0.5 W cm^−2^, and the irradiation time was 10 min. For the 808 nm light treatment, the power density was 1.0 W cm^−2^, and the irradiation time was 10 min. d) Images of representative tumors after treatment with various formulations. e) Time‐dependent body weight of mice under different treatments. f) Histopathological examinations via hematoxylin and eosin (H&E) staining of major organs from mice treated with BM‐PEG NSs. Images were taken under 20× objective.

Given the importance of evaluating the in vivo toxicity of a nanomedicine, the toxicity of BM‐PEG NSs in vivo was assessed in our study through the following methods. First, the immune response following the intravenous injection of BM‐PEG NSs or PBS in healthy mice, including IL‐6, TNF‐α, IL‐12+P40 and IFN‐γ, was analyzed using the serum samples from mice taken 12 and 24 h after injection. The cytokine levels of the BM‐PEG NS treated mice were similar to those of the control group (Figure S20, Supporting Information), indicating no evident cytokine response was induced by the BM‐PEG NSs. In addition, blood routine examination was conducted to measure the amount of lymphocyte (LYM), mean corpuscular hemoglobin concentration (MCHC), mean corpuscular hemoglobin (MCH), red blood cells (RBC), white blood cells (WBC), mean corpuscular volume (MCV), platelet (PLT), creatinine (Cr), neutrophil (NEU), hemoglobin (HGB), and hematocrit (HCT) (Figure S21, Supporting Information). For the histology and hematology assay, the blood of healthy mice that were intravenously treated with BM‐PEG NSs or PBS was collected 1, 7, and 14 days postinjection. Various serum biochemical parameters including aspartate alanine aminotransferase (ALT), aminotransferase (AST), urea nitrogen (BUN), and alkaline phosphatase (ALP) levels were tested. The results are shown in Figure S18 in the Supporting Information. As shown in Figures S20–S22 in the Supporting Information, no statistically significant difference between the BM‐PEG NSs treated group and the control group was observed. The data confirmed the preliminary biosafety of BM‐PEG NSs, which did not induce any infection or inflammation in mice. Additionally, as shown in Figure 5f, there was no obvious tissue damage or inflammation in major organs including the heart, liver, lung, spleen, and kidney.

In summary, we developed a novel methodology to produce ultrathin, exfoliated, BM‐PEG NSs that serve as a robust theranostic platform for multimodal imaging‐guided, photoenhanced CDT/PDT/PTT. By combining grinding, calcination, *n*‐butyllithium exchange and intercalation, and liquid exfoliating processes, ultrathin BM NSs with an average size and thickness of ≈120 and ≈3 nm were fabricated. PEGylated BM NSs exhibited high absorption and photothermal conversion rates of the NIR light, serving as excellent photothermal agents. They efficiently induced hyperthermia following light irradiation at 808 nm. The relatively narrow bandgap (1.45 eV) of BM‐PEG NSs renders them effective photosensitizers that can easily perform electron–hole pairs separation and conversion of O_2_ to ·O_2_^−^ under 650 nm light irradiation, providing efficient PDT for cancer. Moreover, BM‐PEG NSs doped with Fe^2+^ catalyzed the Fenton reaction to generate the most toxic ·OH when H_2_O_2_ was abundant. This reaction could be significantly enhanced upon irradiation by 650 nm light, which produced substantial photoenhanced CDT effects. Additionally, BM‐PEG NSs doped with Fe^3+^ had an ability to modulate the TME through consuming GSH and catalyzing H_2_O_2_ to produce O_2_. The modulation of TME through oxidation of Fe^3+^ not only moderately diminished the antioxidant capability and hypoxia of the tumor, which improved the CDT and PDT efficiency, but also circularly regenerated Fe^2+^ for the Fenton reaction. The ability of BM‐PEG NSs to perform multimodal fluorescent imaging, PA imaging, photothermal imaging, and combination cancer therapy has been demonstrated both in vitro and in vivo.

## Conflict of Interest

The authors declare the following competing financial interest(s): O.C.F. has financial interests in Selecta Biosciences, Tarveda Therapeutics, Placon Therapeutics, and Seer.

## Supporting information

Supplementary
