# Supplementary material for: Synthesis of Ultrathin Biotite Nanosheets as an Intelligent Theranostic Platform for Combination Cancer Therapy
Source: Adv Sci (Weinh). 2019 Aug 20;6(19):1901211. doi: 10.1002/advs.201901211 (PMC6774039; doi:10.1002/advs.201901211)
Supplement: Supplementary file 1 — Supplementary [file ADVS-6-1901211-s001.pdf]

Copyright WILEY-VCH Verlag GmbH & Co. KGaA, 69469 Weinheim, Germany, 2019.

ADVANCED SCIENCE
Open Access

Supporting Information

for *Adv. Sci.,* DOI: 10.1002/advs.201901211

Synthesis of Ultrathin Biotite Nanosheets as an Intelligent Theranostic Platform for Combination Cancer Therapy

Xiaoyuan Ji, Yong Kang, Jiang Ouyang, Yunhan Chen, Dolev Artzi, Xiaobin Zeng, Yuling Xiao, Chan Feng, Baowen Qi, Na Yoon Kim, Phei Er Saw, Na Kong, Omid C. Farokhzad,* and Wei Tao**

Synthesis of Ultrathin Biotite Nanosheets as an Intelligent Theranostic Platform for Combination Cancer Therapy

Xiaoyuan Ji, Yong Kang, Jiang Ouyang, Yunhan Chen, Dolev Artzi, Xiaobin Zeng, Yuling Xiao, Chan Feng, Baowen Qi, Na Yoon Kim, Phei Er Saw, Na Kong, Omid C. Farokhzad*, Wei Tao**

Dr. X. Ji, Dr. X. Zeng
Center Lab of Longhua Branch, and Department of Infectious Disease, Shenzhen People's Hospital, 2nd Clinical Medical College of Jinan University, Shenzhen 518120, Guangdong Province, China.
E-mail: zengxiaobin1983@163.com

Dr. X. Ji
Integrated Chinese and Western Medicine, Postdoctoral research station, Jinan University, Guangzhou 510632, China

Dr. X. Zeng
Guangdong Provincial Key Laboratory of Regional Immunity and Diseases, School of medicine, Shenzhen University

Dr. X. Ji, J. Ouyang, Y. Chen, D. Artzi, Dr. Y. Xiao, C. Feng, Dr. B. Qi, N.Y. Kim, Dr. P. Saw, Dr. N. Kong, Prof. O. C. Farokhzad, Dr. W. Tao
Center for Nanomedicine
Brigham and Women's Hospital
Harvard Medical School
Boston, MA 02115, USA
E-mail: wtao@bwh.harvard.edu
E-mail: ofarokhzad@bwh.harvard.edu

Y. Kang
State Key Laboratory of Biochemical Engineering
Institute of Process Engineering, Chinese Academy of Sciences
Beijing 100190, China

Materials.

Natural black mica (BM) was supplied by Lingshou Huaguan Co. Ltd. (Lingshou, Hebei, China). Cy7-PEG-NH_2 (MW: 5k) and PEG-NH_2 (MW: 5k) were supplied by Nanocs Inc. PI and Calcein-AM were bought from Invitrogen (USA). RPMI medium, DMEM medium, fetal bovine serum (FBS), trypsin-EDTA, and PBS (pH 7.4) were purchased from Gibco Life Technologies. H_2O_2 (30%), glutathione, 5,5′-dithiobis (2-nitrobenzoic acid) (DTNB), 1,3-diphenylisobenzofuran (DPBF), methylene blue (MB), and [Ru(dpp)$_3$]Cl_2 (RDPP) were purchased from Sigma-Aldrich.

Preparation of BM Nanosheets (NSs).

BM NSs were prepared by combining grind, calcination, ionic liquid intercalation, and liquid-phase exfoliation. First, commercial BM powder was dispersed in NMP solution with the initial concentration of 50 mg/mL. The solution was ground for 1 h at 1000 rpm. After drying, the ground BM was calcined at 800 ^oC for 2 h in a furnace under nitrogen atmosphere. After cooling, the calcined BM was immersed in n-butyllithium solution (20 mL, 1.6 M) under nitrogen atmosphere and heated at 50 ^oC for 48 h. Next, the excess n-butyllithium was removed by centrifuging and washing three times. The intercalated BM was then immersed in 2 mg/mL NMP solution and ultrasonicated for 12 h. After the exfoliation, the BM solution was then centrifuged at 3000 rpm to remove the unexfoliated BM. The supernatant was centrifuged at 12000 rpm and washed three times with DI water to eliminate NMP. The obtained BM NSs were dispersed in PBS and stored at 4 ^oC.

PEG Coating on the Surface of BM NSs.

In order to prepare PEGylated BM NSs, PEG-NH_2 (10 mg) was dissolved in BM NSs solution (100 μg/mL, 10 mL). After probe sonication and stirring for 30 min and 12 h respectively, the resulting mixture was centrifuged in Amicon tubes (MWCO 100kDa; Millipore) at 2500 rpm (4 °C) for 30 min, and washed three times to remove the unattached PEG-NH_2 molecules. The PEGylated BM NS sample was resuspended in PBS for further use and stored at 4 °C. For the fluorescent modification of B-PEG-Cy7 NSs, the same procedures were performed in the dark, and Cy7-PEG-NH_2 was used instead of PEG-NH_2.

Characterization.

The morphology and thickness of BM NSs and BM-PEG NSs were characterized *via* transmission electron microscopy (TEM, JEM-2100UHR, JEOL, Japan) and atomic-force microscopy (AFM, FASTSCANBIO, Germany), respectively. The surface charge (zeta potential) and size of BM NSs

were detected by Dynamic Light Scattering. The chemical compositions of the NSs were analyzed by Fourier transform infrared spectrophotometry (FTIR, Nexus 470, Nicolet, Madison, WI, USA) and X-ray photoelectron spectroscopy (XPS, ESCALAB 250Xi, Japan). An energy-dispersive X-ray spectroscope (EDS) (Inca X-MAX, Oxford, UK) was used to analyze the elementary composition of NSs. UV-Vis-NIR spectra of NSs were obtained on an Infinite M200 PRO spectrophotometer. The chemical structures of the NSs were analyzed by X-ray diffraction (XRD) patterns, performed on a Bruker D8 multipurpose XRD system.

Photothermal Performance of BM-PEG NSs.

To investigate the photothermal conversion of BM-PEG NSs, aqueous BM-PEG NS dispersions at different concentrations (50-200 μg/mL, 1 mL) were irradiated by an 808-nm NIR laser at a power density of 1, 1.5, and 2 W cm^{-2} for 5 min. The temperature of the BM-PEG NS solution was recorded using an IR thermal camera every 30 s.

Photothermal Conversion Efficiency Calculation.

The method to calculate the photothermal conversion efficiency of BM-PEG NSs is shown below:

Based on the total energy balance for this system:

$$\sum_i m_i C_{p,i} \frac{dT}{dt} = Q_{BM\ NSs} + Q_s - Q_{loss} \quad (1)$$

where T is the solution temperature. C_p and m are the heat capacity and mass of solvent (water), respectively.

$Q_{BM\ NSs}$ is the photothermal energy investment by BM-PEG NSs:

$$Q_{BM\ NSs} = I(1 - 10^{-A_{808}})\eta \quad (2)$$

where I is the laser power, A_{808} is the absorbance of BM-PEG NSs at 808 nm, and η is the conversion efficiency.

Q_s is the heat related to the photo absorbance of water.

Q_{loss} is the thermal energy lost to the environment:

$$Q_{loss} = hA\Delta T \quad (3)$$

h is the heat transfer coefficient, ΔT is the temperature change, and A is the surface area of the container.

At the maximum steady-state temperature, the heat input is same as the heat output, that is:

$$Q_{BM\ NSs} + Q_s = Q_{loss} = hA\Delta T_{max} \quad (4)$$

ΔT_{max} is the temperature change at the maximum steady-state temperature. According to the Eq.2 and Eq.4, the η can be caculated as follows:

$$\eta = \frac{hA\Delta T_{max} - Q_s}{I(1-10^{-A_{808}})} \tag{5}$$

For the sake of the value of *hA*, the ratio of ΔT to ΔT_{max}, θ, was introduced:

$$\theta = \frac{\Delta T}{\Delta T_{max}} \tag{6}$$

Substituting Eq.7 into Eq.2 and rearranging Eq.2:

$$\frac{d\theta}{dt} = \frac{hA}{\sum_i m_i C_{p,i}}\left[\frac{Q_{BM\ NSs} + Q_s}{hA\Delta T_{max}} - \theta\right] \tag{7}$$

When the laser was shut off, QNSs+Qs = 0, so Eq.8 changed to:

$$dt = -\frac{\sum_i m_i C_{p,i}}{hA}\frac{d\theta}{\theta} \tag{8}$$

Integrating Eq.8 gives the expression:

$$t = -\frac{\sum_i m_i C_{p,i}}{hA}\theta \tag{9}$$

Thus, the η of BM-PEG NSs can be calculated through determining *hA* and substituting to Eq. 6.

Extracellular GSH Depletion

A GSH and DTNB solution with the final concentration of 0.1 mM GSH and 0.2 mg/mL DTNB was prepared and maintained at 25 °C. The BM-PEG NS solutions with the final concentrations of 25, 50, 100, 200 μg mL^{-1} were added to the above solution and reacted for 30 min. Every 5 min, the reaction solution was centrifuged at 10000 rpm to precipitate BM-PEG NSs, and UV−vis spectroscopy was applied to detect the absorbance of the supernatant.

Extracellular O_2 Production

H_2O_2 with a final concentration of 0.1 mM was added to the BM-PEG NS solution (200 mg mL^{-1}, 10 mL). The purchased oxygen meter was used to measure the generated O_2 according to the manufacturer’s instructions.

Extracellular ·OH Measurement.

MB was used to assess the capability of BM-PEG NSs to generate ·OH in the aqueous solution. Briefly, 1.5 mL of DI water was used to dissolve MB dye (10 mg m L^{-1}) and BM-PEG NSs (200 mg mL^{-1}). Before the reaction. the solution was stirred in a dark room for 1 h to reach the adsorption/desorption equilibrium. Then, H_2O_2 with the final concentration of 0.1 mM was added and irradiated with a 0.5 W cm^{-2} 650 nm laser for 30 min. After the irradiation, the MB solution was

centrifuged to precipitate BM-PEG NSs. UV−vis spectroscopy was applied to detect the absorbance of the supernatant.

Extracellular $\cdot O_2^-$ Detection.

The detection of the extracellularly generated $\cdot O_2^-$ was carried out using DPBF as a probe of $\cdot O_2^-$. 1.5 mL deionized aqueous solution containing DPBF dye (1 mM) and BM-PEG NSs (200 mg mL^{-1}) was mixed with H_2O_2 (10 mM, 15 μL). Then the mixture was irradiated by a 650 nm laser (0.5 W cm^{-2}) for 30 min. Every 5 min, the reaction solution was centrifuged at 10000 rpm to precipitate BM-PEG NSs, and UV−vis spectroscopy was applied to detect the absorbance of the supernatant.

Cytotoxicity of BM-PEG NSs.

HeLa, PC3, MCF7, and A549 cells cells were cultured in 96-well plates for 24 h (37 °C, 5% CO_2). Subsequently, BM-PEG NSs (25, 50, 100, 200, and 300 μg mL^{-1}) were added to the above cells for another 24 h. Finally, the cells were washed with PBS three times and the viabilities of the cells were determined by AlamarBlue assay (Life Technologies).

Intracellular GSH Depletion

The intracellular depletion of GSH was detected using the GSH assay kit. MCF7 cells were cultured in 96-well plates for 24 h (37 °C, 5% CO_2). Then, fresh culture medium that contained pre-dissolved BM-PEG NSs (50 μg/mL) was used to replace the old culture medium, and incubation was continued for another 24 h. Subsequently, the treated cells were rinsed with PBS and centrifuged to collect the sediment. After that, the collected cells were crushed using an ultrasound cell crusher in 1 mL of PBS. Finally, the concentration of intracellular GSH was determined by the GSH assay kit.

Intracellular O_2 Generation

The intracellular production of O_2 was tested using RDPP as the O_2 probe by CLSM imaging. MCF7 cells were seeded on culture dishes and cultured for 24 h (37 °C, 5% CO_2). Then, fresh culture medium that contained pre-dissolved RDPP (1 μM) was used to replace the old culture medium, and incubation was continued for 4 h. Then, the MCF7 cells were washed with PBS and incubated with fresh culture medium that contained pre-dissolved BM-PEG NSs (200 μg mL^{-1}) for another 24 h. Finally, the cells were washed for three times by PBS, and detected by CLSM.

Intracellular ROS Production

The ROS probe, DCFH-DA, was applied to analyze the intracellular generation of ROS *via* CLSM.

MCF7 cells were cultured in culture dishes for 24 h (37 °C, 5% CO_2). Subsequently, fresh culture medium that contained pre-dissolved BM-PEG NSs (200 μg mL^{-1}) was used to replace the old culture medium, and incubation was continued for another 24 h. Then, DCFH-DA solution with a concentration of 0.2 μM was added and the cells were cultured for 0.5 h. Subsequently, the cells were washed three times by PBS and irradiated by a 650 nm laser (0.5 W cm^{-2}) for 10 min. The green fluorescence of ROS was detected by CLSM.

***In vitro* Photothermal, Photodynamic, and Photoenhanced Chemodynamic Therapy.**

MCF7 and PC3 cells were cultured in 96-well plates for 24 h (37 °C, 5% CO_2). Then, fresh culture medium that contained pre-dissolved BM-PEG NSs (0, 25, 50, 100, and 200 μg mL^{-1}) was used to replace the old culture medium, and incubation was continued for another 24 h. Then, the cells were washed three times by PBS and re-cultured with fresh medium. After that, cells were exposed to an 808 nm laser (1.0 W cm^{-2}) or 650 nm laser (0.5 W cm^{-2}) for 10 min, respectively. After 24 h of cell culture, the cells were washed three times by PBS and cell viability was determined by AlamarBlue assay.

Pharmacokinetic Study.

For the pharmacokinetic study *in vivo*, healthy C57BL/6 mice were *i.v.* injected with 200 μL of Cy7-PEG-NH_2 loaded BM NSs ([BM-PEG NSs]= 6 mg/kg). At different time intervals, 20 μL blood was collected. BioTekmicroplate reader was used to measure the fluorescence of Cy7 in blood.

Xenograft Tumor Model.

MCF7 cells were suspended in 200 μL cell medium and implanted in nude Balb/c mice subcutaneously. After the tumor had grown to ~100 mm^3, the mice were used for *in vivo* imaging and antitumor experiments.

***In vivo* Fluorescence Imaging and Biodistribution Study.**

For fluorescence imaging and biodistribution analysis of the NSs *in vivo*, MCF7 tumor-bearing mice were given an intravenous injection of Cy7-PEG-NH_2 loaded BM-PEG NSs *via* tail vein. Maestro2 In-Vivo Imaging System was used to detect the fluorescence at 24 h post-injection. Furthermore, Maestro2 In-Vivo Imaging System was used to image major organs and tumors. The content of NSs in tumors and major organs was quantified through measuring the fluorescence intensity of Cy7 by Image-J. The intensity values were then divided by the weight (g) of each organ.

***In vitro* and *in vivo* Photoacoustic Imaging.**

For PA imaging *in vitro*, BM-PEG NSs in DI water at different concentrations, ranging from 0 to 4.0 mg/mL), were used for PA signal measurement. The linear relationship between the PA signal and BM-PEG NS concentration was detected by the Vevo LAZR photoacoustic imaging system (Visual-Sonics Co.)

For *in vivo* PA imaging, BM-PEG NSs were intravenously injected to MCF7 tumor-bearing mice. At 12 h and 24 h post-injection, the Vevo LAZR photoacoustic imaging system (Visual-Sonics Co.) was used to record the PA signal of MCF7 tumor-bearing mice.

***In vivo* Photothermal, Photodynamic, and Photoenhanced Chemodynamic Therapy.**

For *in vivo* combined therapy, 6 treatment groups with 5 mice for each group were randomly divided when the tumor size reached ~100 mm^3. Group 1: saline (control group), Group 2: 650 + 808 nm laser (NIR laser group), Group 3: BM-PEG NSs (CDT group) ([BM-PEG NSs]= 6 mg/kg), Group 4: BM-PEG NSs + 650 nm laser (photoenhanced CDT/PDT group), Group 5: BM-PEG NSs + 808 nm laser (PTT group) and Group 6: BM-PEG NSs + 650 + 808 nm (photoenhanced CDT/PDT/PTT group). For the 650nm laser treatment, the power density was 0.5 W cm^{-2}, and the irradiation time was 10 mins. For the 808nm laser treatment, the power density was 1.0 W cm^{-2}, and the irradiation time was 10 mins. For group 2 and 6, the mice were successively irradiated (808 nm and 650 nm) at 24 h postinjection. Group 4 was exposed to a 650nm laser and Group 5 was exposed to a 808 nm laser. During treatment, the tumor size of each group was recorded every two days for 14 days and calculated as volume.

***In vivo* Toxicity.**

For the *in vivo* toxicity study, BM-PEG NSs in PBS ([BM-PEG NSs]= 10 mg/kg) was intravenously injected into healthy C57BL/6 mice. 30 days after injection, the organs were harvested and used for hematoxylin and eosin (H&E) staining.

For the analysis of the immune response, BM-PEG NSs in PBS ([BM-PEG NSs]= 10 mg/kg) was intravenously injected into healthy C57BL/6 mice. 12 h and 24 h after injection, the concentrations of interleukin 6 (IL-6), tumor necrosis factor-α (TNF-α), interleukin-12 (IL-12+P40), and interferon-γ (IFN-γ) were detected by ELISA. For the complete blood panel test, lymphocyte (LYM), mean corpuscular hemoglobin concentration (MCHC), mean corpuscular hemoglobin (MCH), red blood cells (RBC), white blood cells (WBC), mean corpuscular volume (MCV), platelet (PLT), creatinine (Cr), neutrophil (NEU), hemoglobin (HGB) and hematocrit (HCT) at 1, 7, and 14 d after

intravenous injection were measured. For the blood chemistry analysis, alanine aminotransferase (ALT), aspartate aminotransferase (AST), urea nitrogen (BUN) and alkaline phosphatase (ALP) at 1, 7, and 14 d after intravenous injection were also measured.

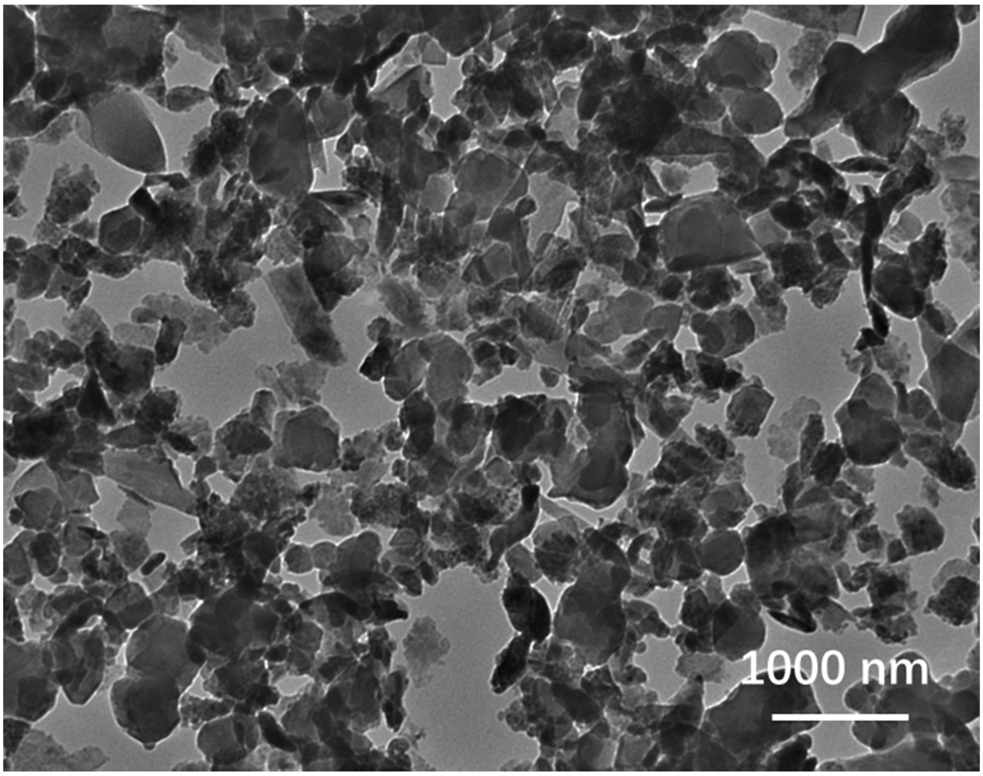

Figure S1. TEM image of grinded BM.

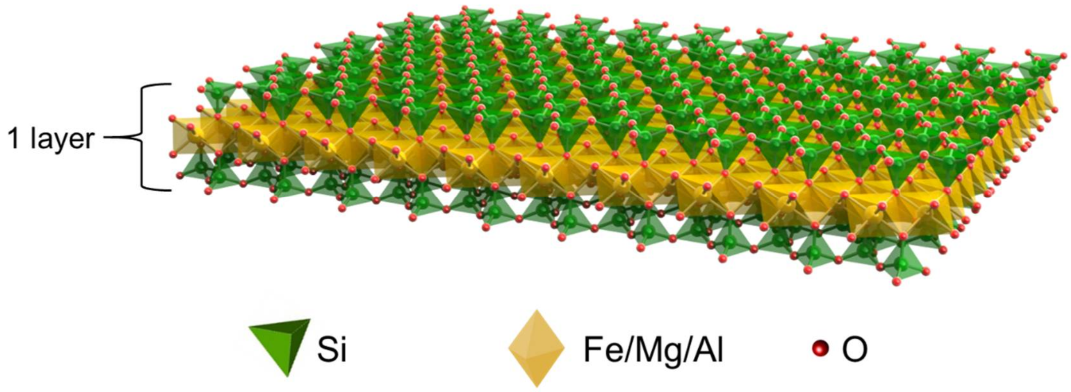

Figure S2. The atomic structure of single layer BM NSs.

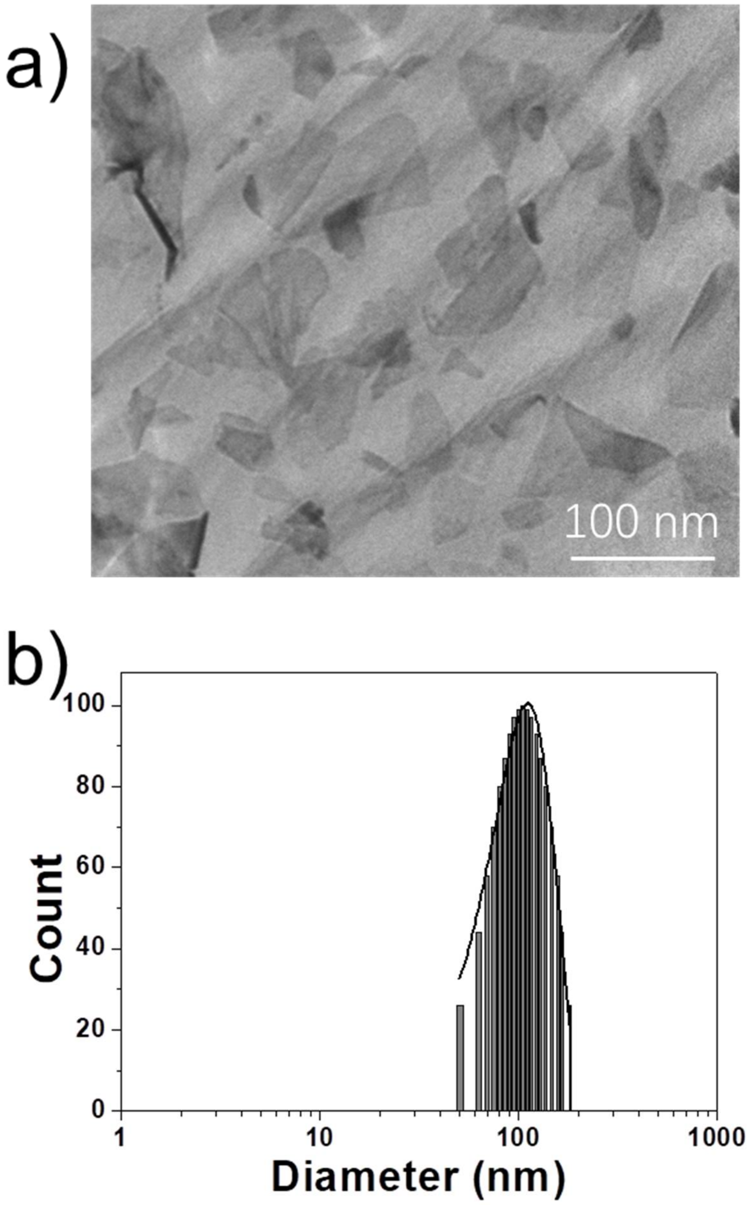

Figure S3. (a) TEM image and (b) size distribution of BM NSs.

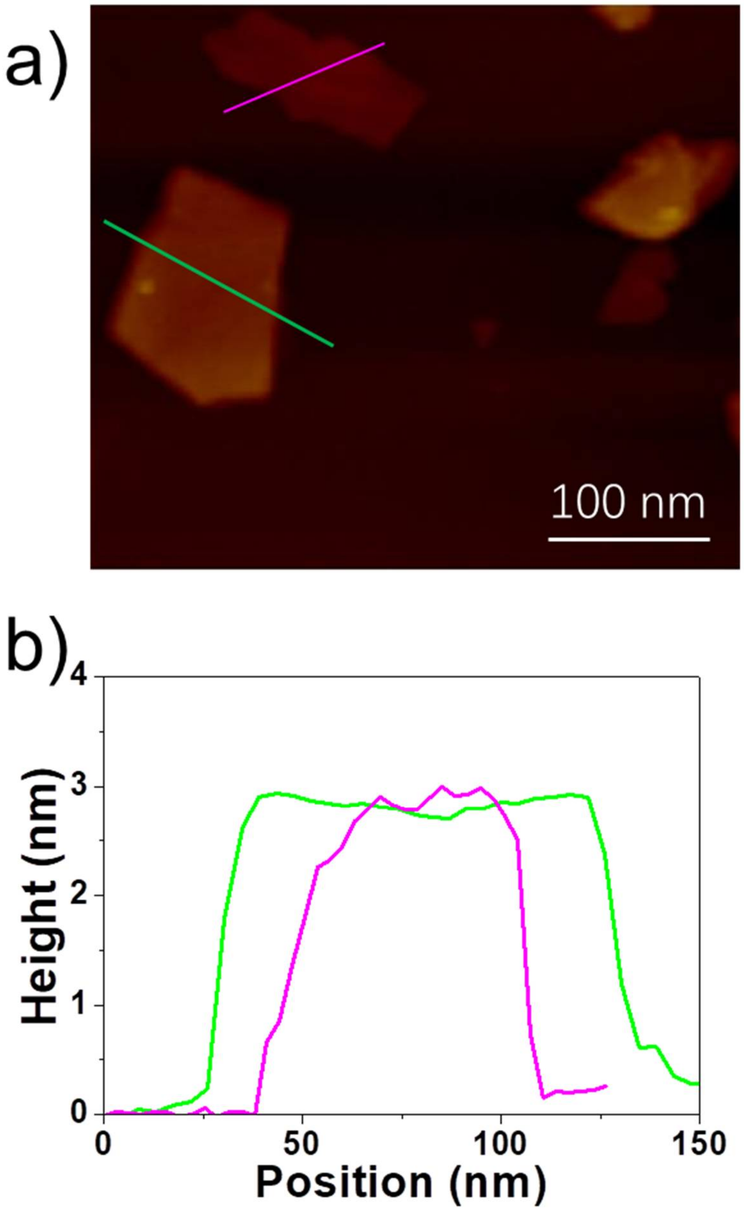

Figure S4. (a) AFM images and (b) thickness of BM NSs.

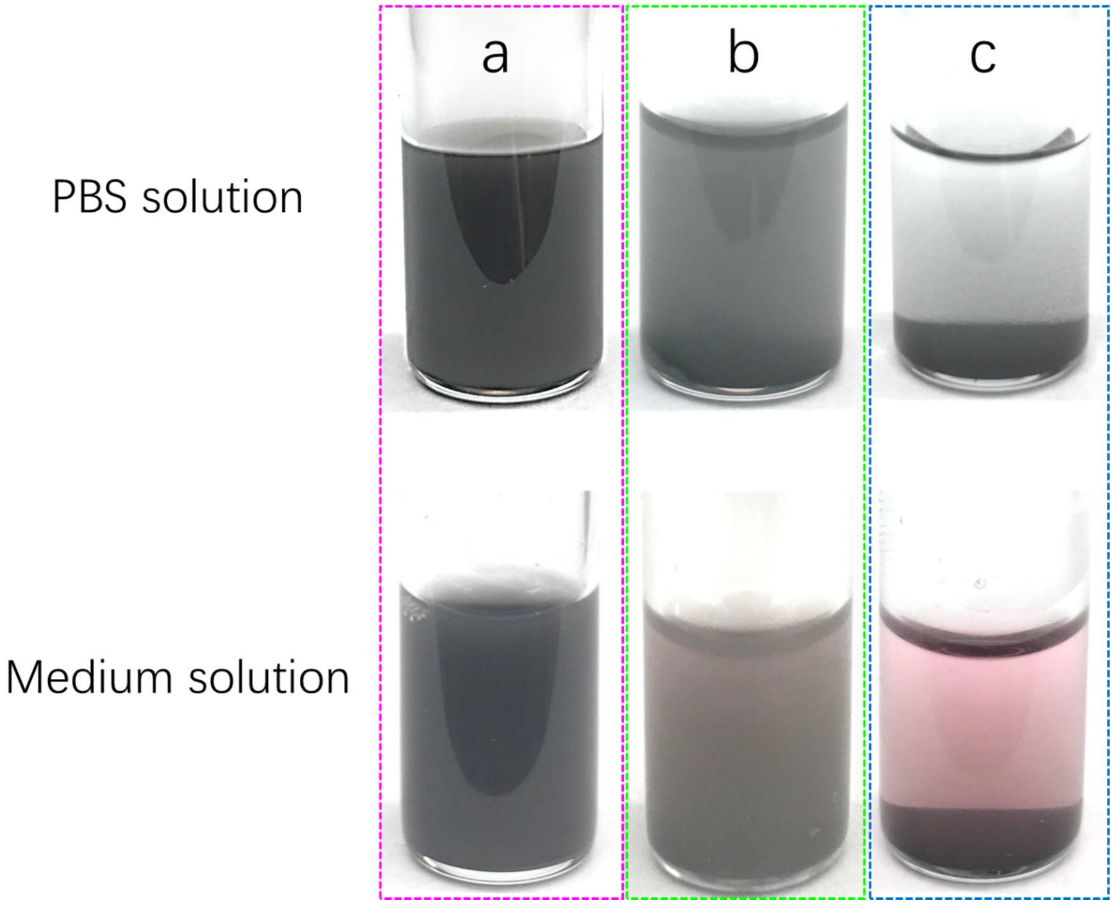

Figure S5. The dispersion of the BM-PEG NSs in different solutions (PBS and cell culture medium) after 24 h: a) well-prepared, b) thicker, and c) larger BM-PEG NSs.

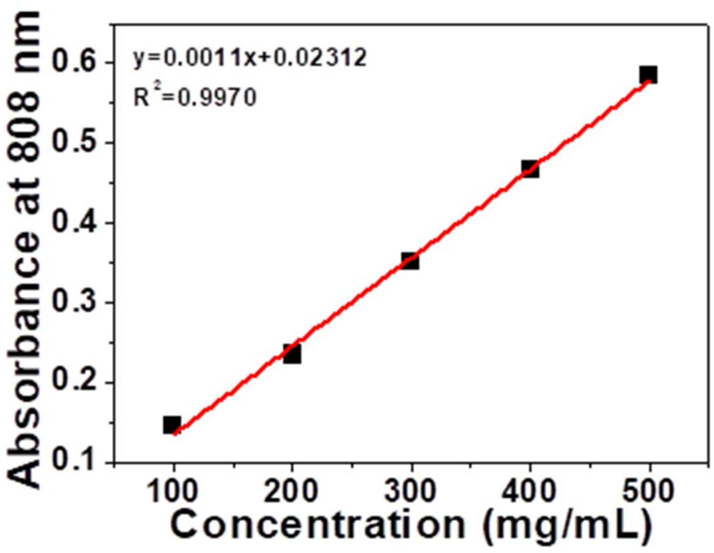

Figure S6. Normalized absorbance intensity of BM-PEG NSs at 808 nm.

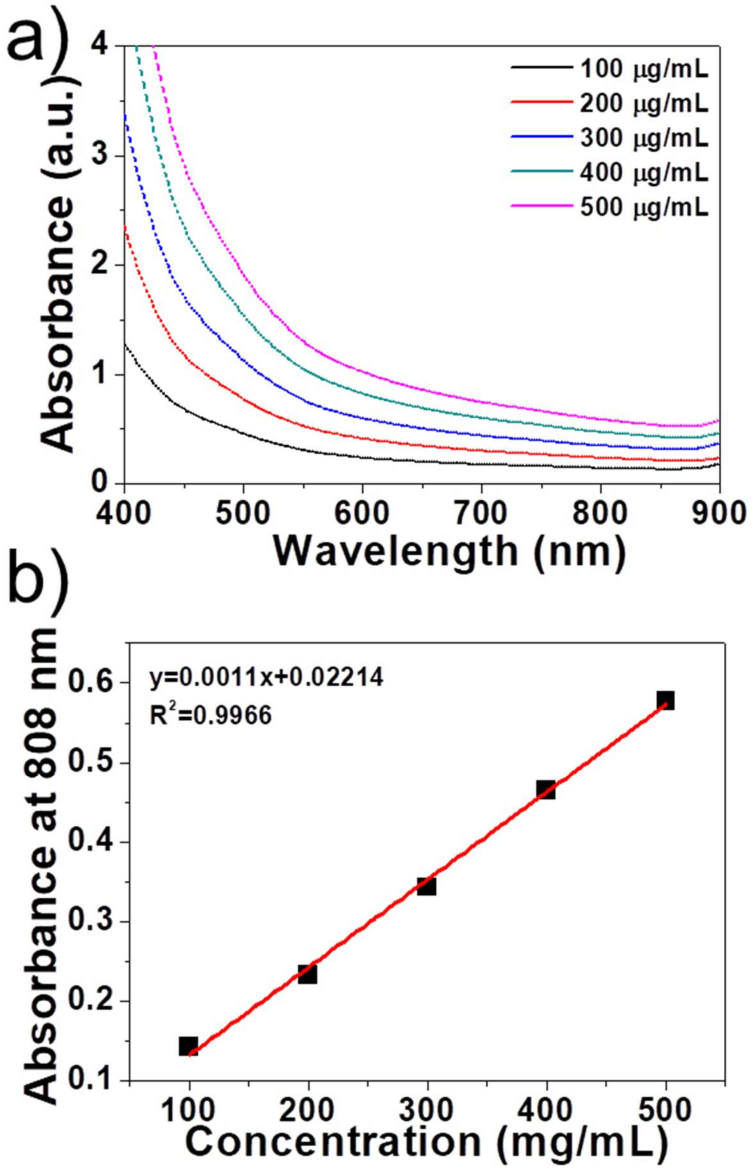

Figure S7. (a) Absorbance of BM NSs. (b) Normalized absorbance intensity of BM NSs at 808 nm.

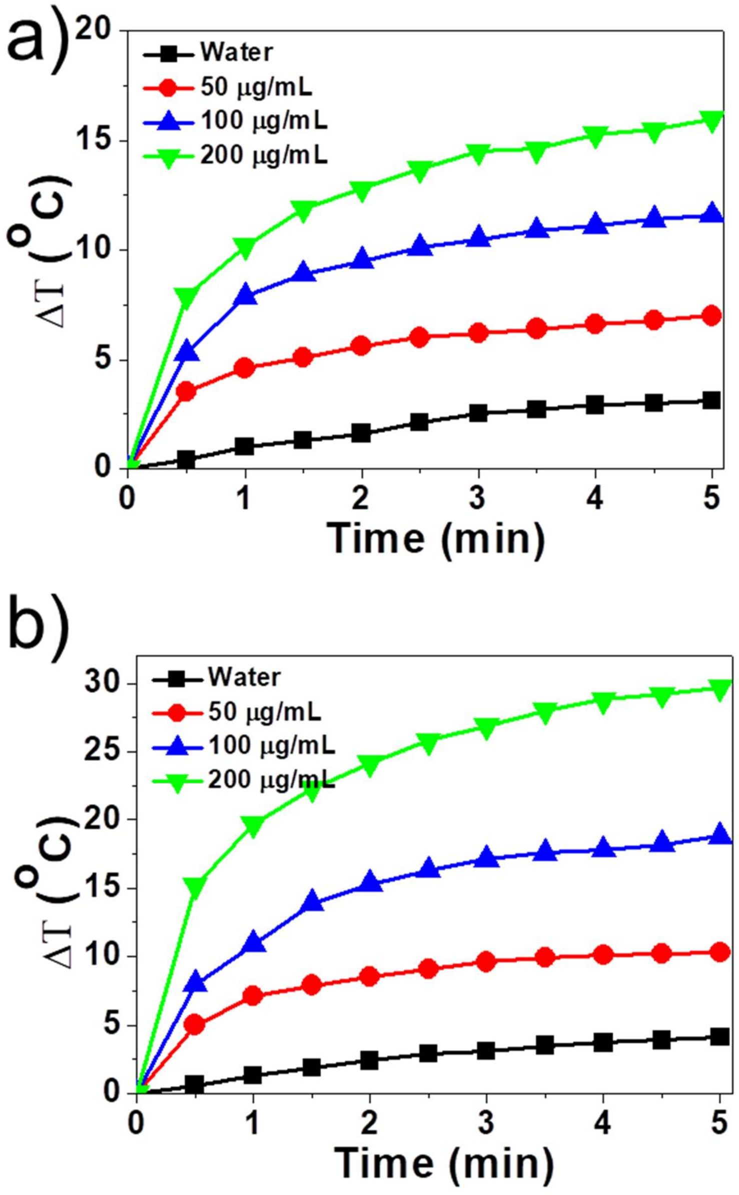

Figure S8. a) and b) Temperature changes of different concentrations of BM-PEG NSs solutions as a function of irradiation time - 5 min exposure to 808 nm laser at 1 W/cm^2 and 1.5 W/cm^2.

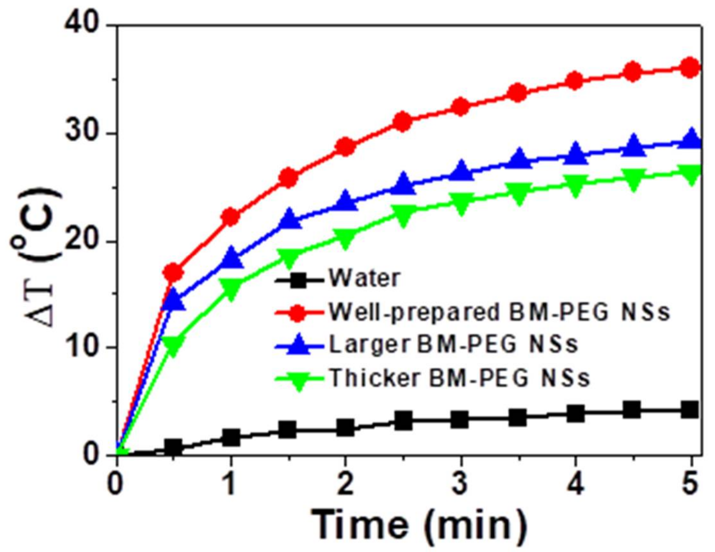

Figure S9. Temperature change curves of BM-PEG NSs with different size and thickness exposed to an 808 nm laser (2 W cm^{-2}) for 5 min.

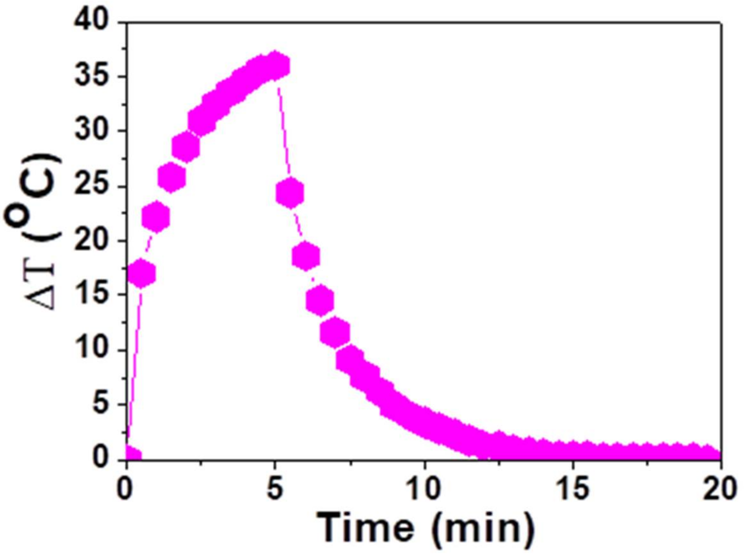

Figure S10. Temperature changes of different concentrations of BM-PEG NSs solutions as a function of irradiation time - 5 min exposure to 808 nm laser at 2 W cm^{-2}.

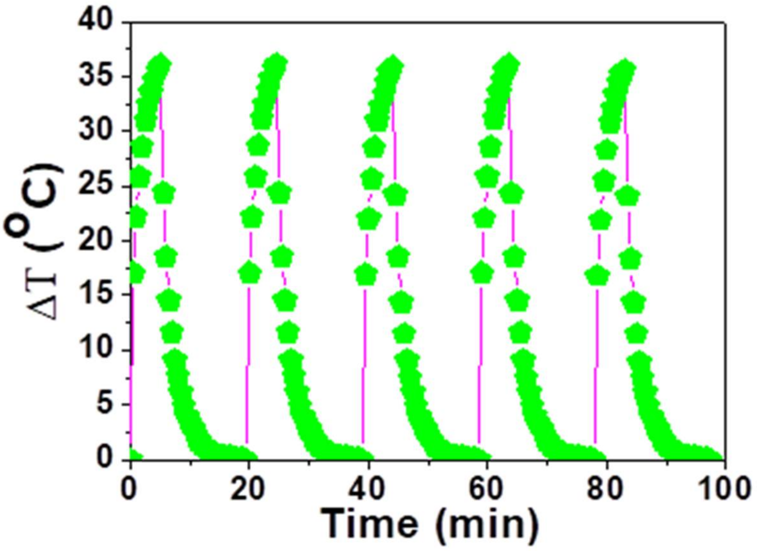

Figure S11. Heating and cooling curves of BM-PEG NSs (200 μg mL^{-1}) solution at power density of 2 W cm^{-2}.

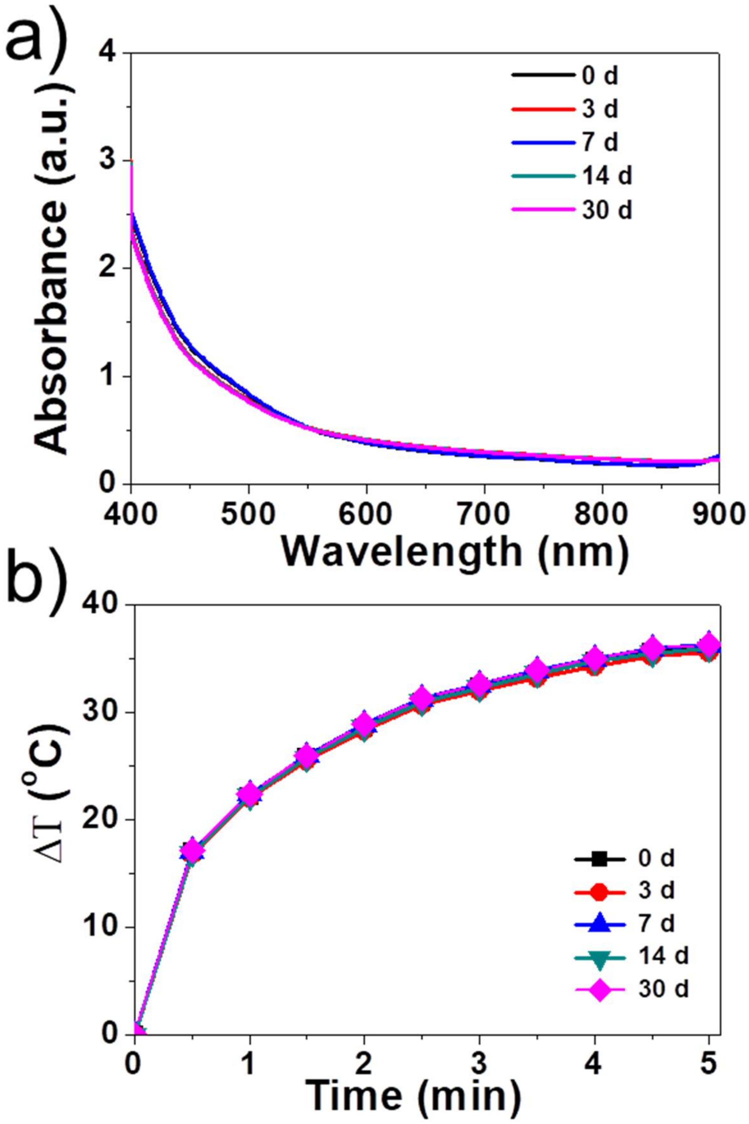

Figure S12. (a) The photo absorbance and (b) photothermal conversion curves of BM-PEG NSs after storing for 30 days.

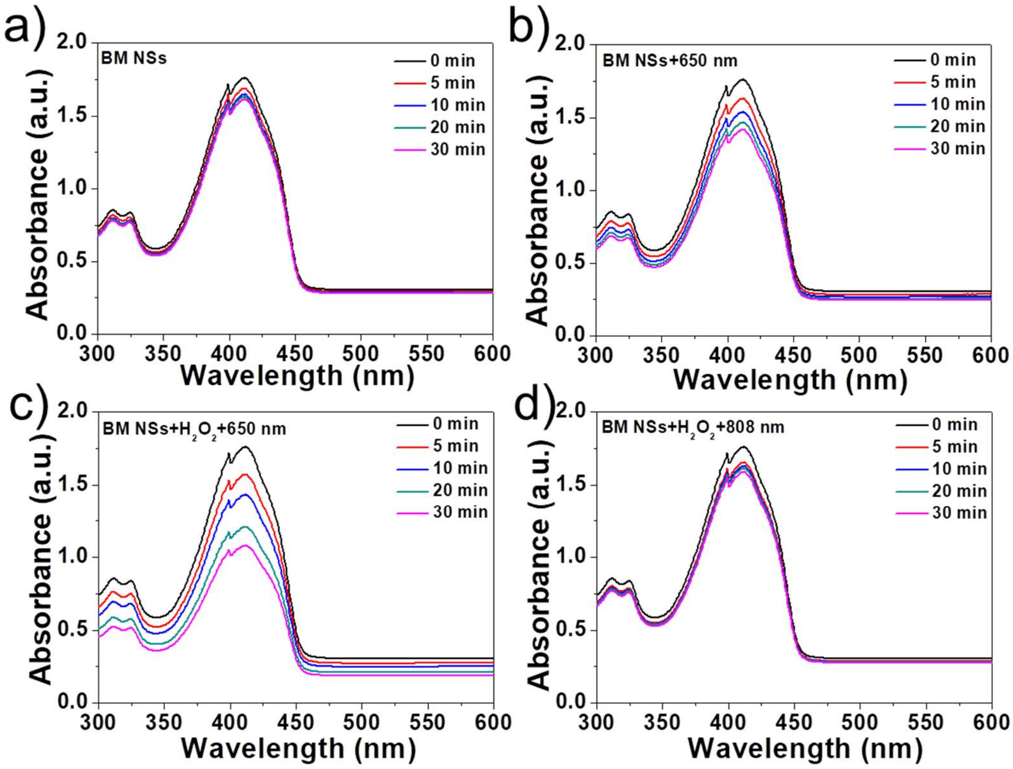

Figure S13. Degradation of DPBF *via* generated $\cdot O_2^{-}$: (a) BM-PEG NSs, (b) BM-PEG NSs + 650 nm laser, (c) BM-PEG NSs + H_2O_2 + 650 nm laser, and (d) BM-PEG NSs + H_2O_2 + 808 nm laser. The concentrations of BM-PEG NSs and H_2O_2 were 200 μg mL^{-1} and 250 μM respectively. The power density of 650 nm and 808 nm lasers were 0.5 and 2 W cm^{-2}.

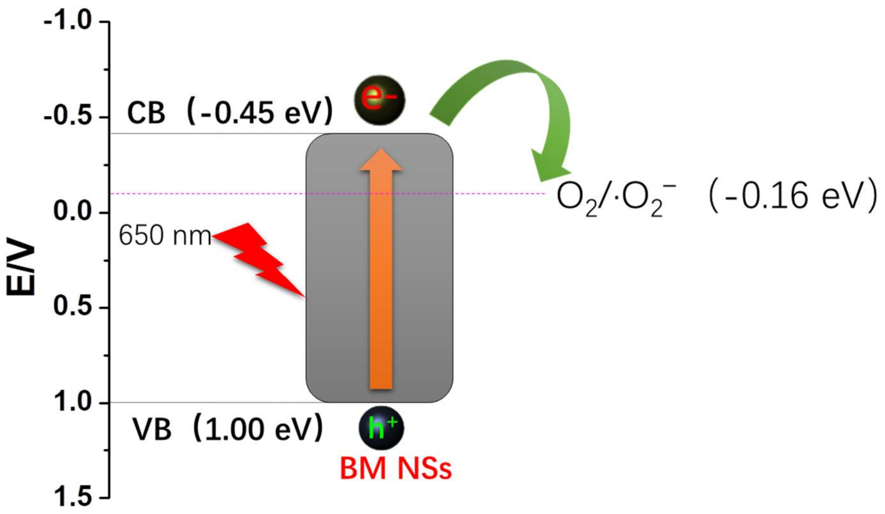

Figure S14. The schematic illustration of PDT (type I) based on BM-PEG NSs.

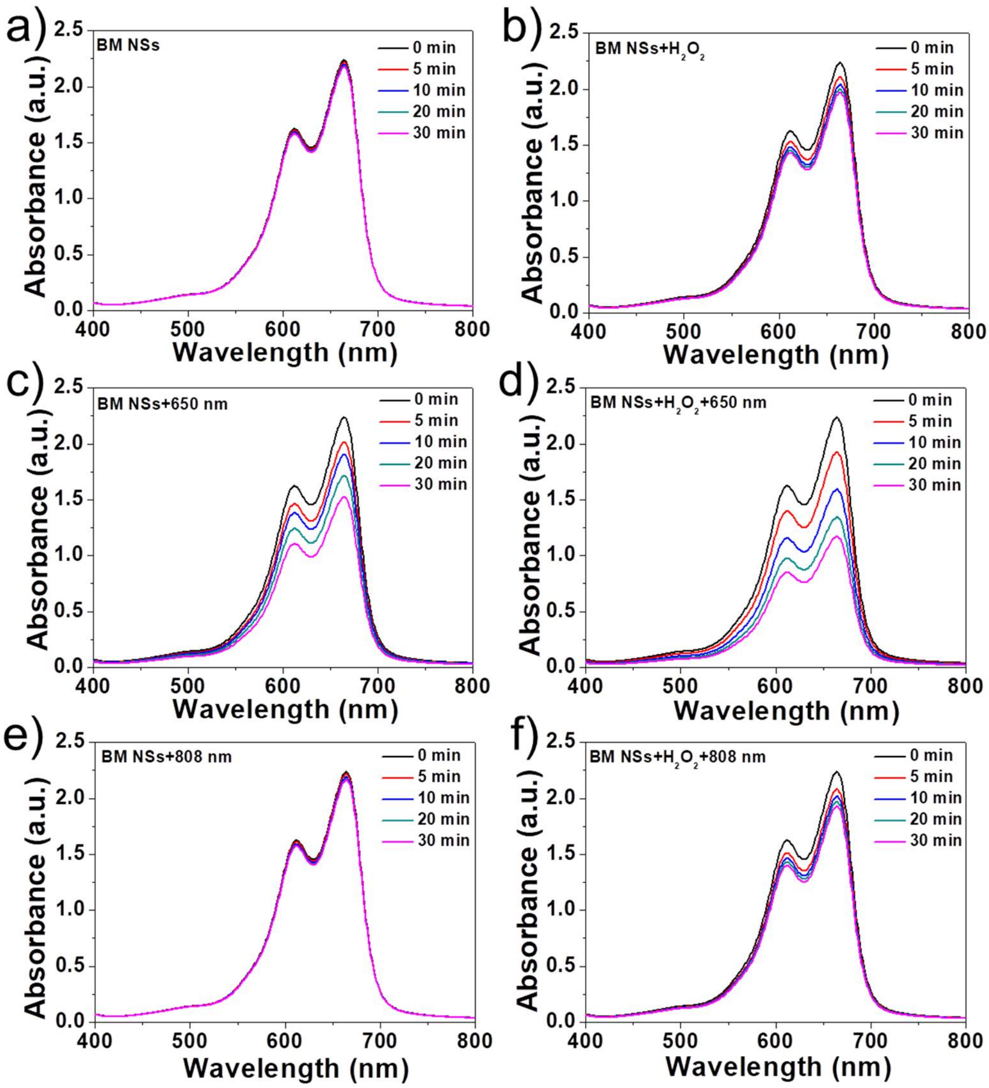

Figure S15. Degradation of MB *via* generated ·OH: (a) BM-PEG NSs, (b) BM-PEG NSs + H_2O_2, (c) BM-PEG NSs + 650 nm laser, (d) BM-PEG NSs + H_2O_2 + 650 nm laser, (e) BM-PEG NSs + 808 nm laser, and (f) BM-PEG NSs + H_2O_2 + 808 nm laser. The concentrations of BM-PEG NSs and H_2O_2 were 200 μg mL^{-1} and 250 μM respectively. The power density of 650 nm and 808 nm lasers were 0.5 and 2 W cm^{-2}.

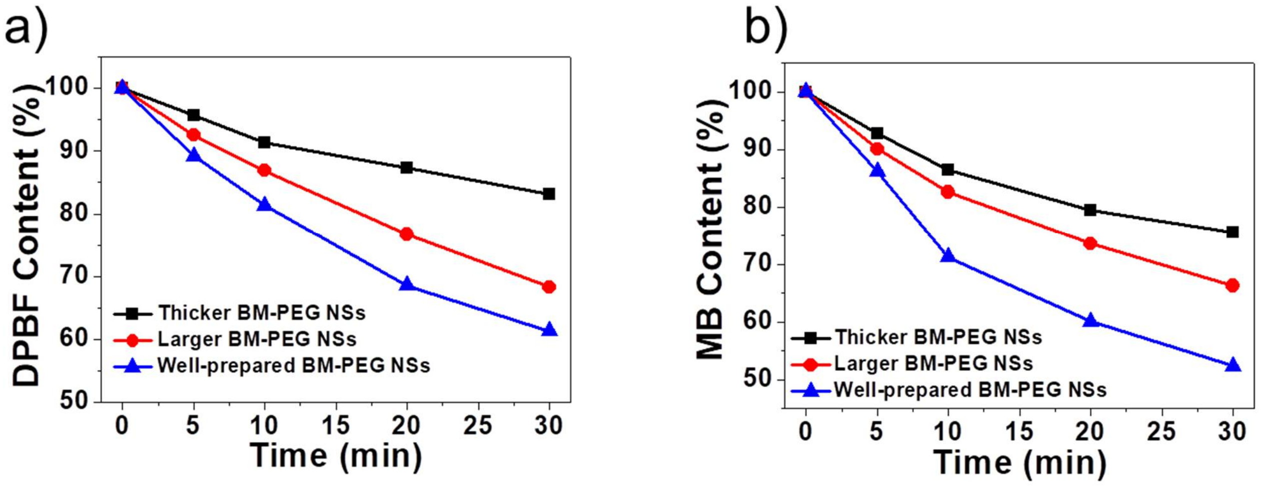

Figure S16. a) Degradation of DPBF owing to $\cdot O_2^-$ generation after the treatment of different BM-PEG NSs with different size and thickness. b) Depletion of MB owing to ·OH production after the treatment of different BM-PEG NSs with different size and thickness.

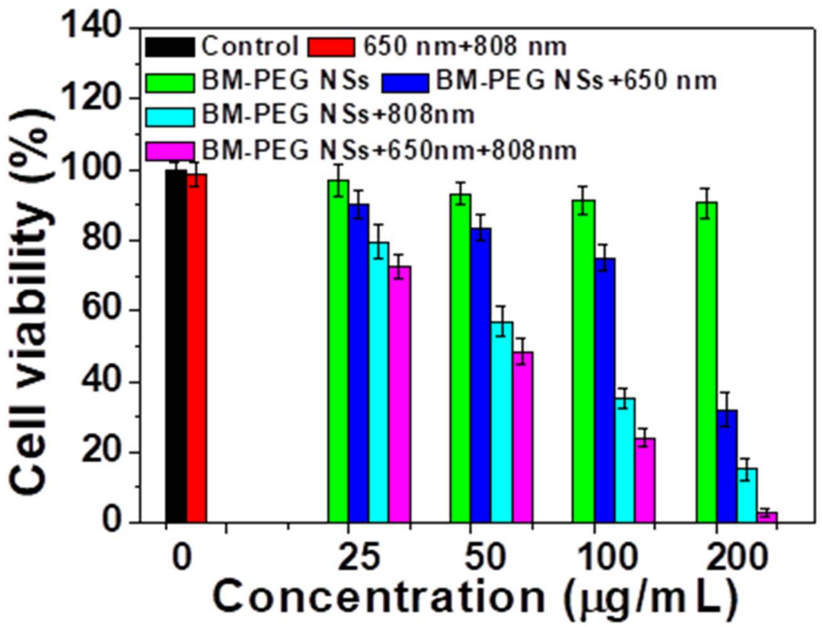

Figure S17. PC3 viability under different treatments. For the 650 nm light treatment, the power density was 0.5 W cm^{-2}, and the irradiation time was 10 min. For the 808 nm light treatment, the power density was 1.0 W cm^{-2}, and the irradiation time was 10 min.

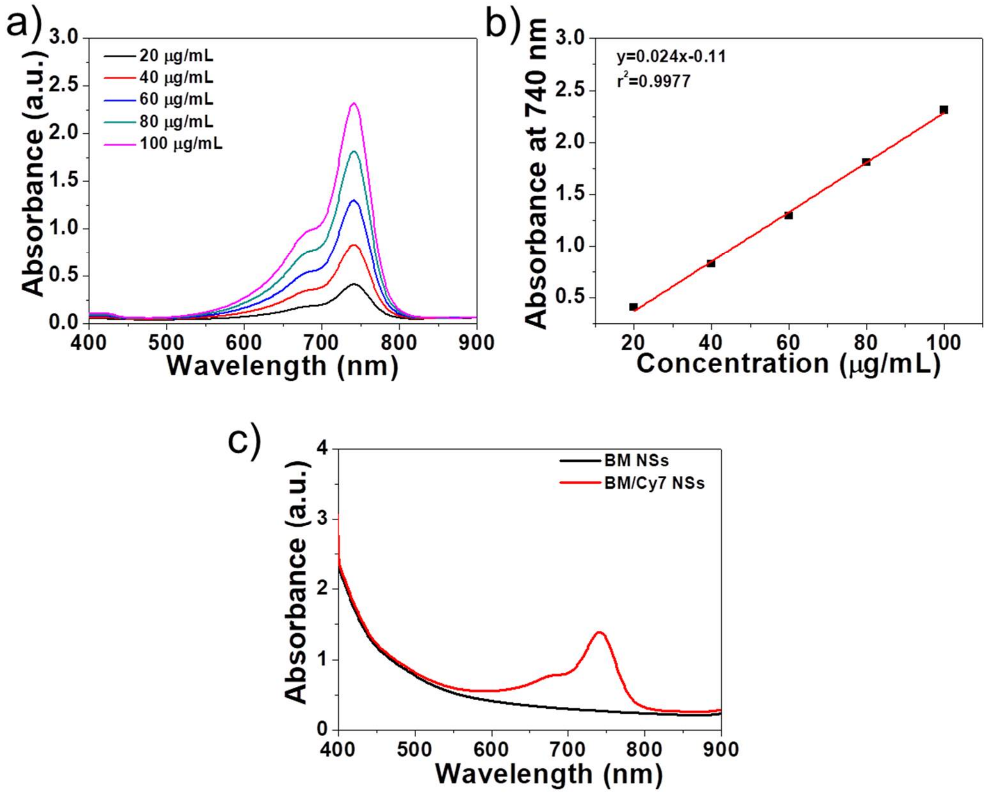

Figure S18. (A) The absorbance of free Cy7-PEG-NH_2. (b) Normalized absorbance intensity of Cy7-PEG-NH_2 at different concentrations for λ=740 nm. (c) The absorbance of BM-PEG NSs and BM-PEG-Cy7 NSs.

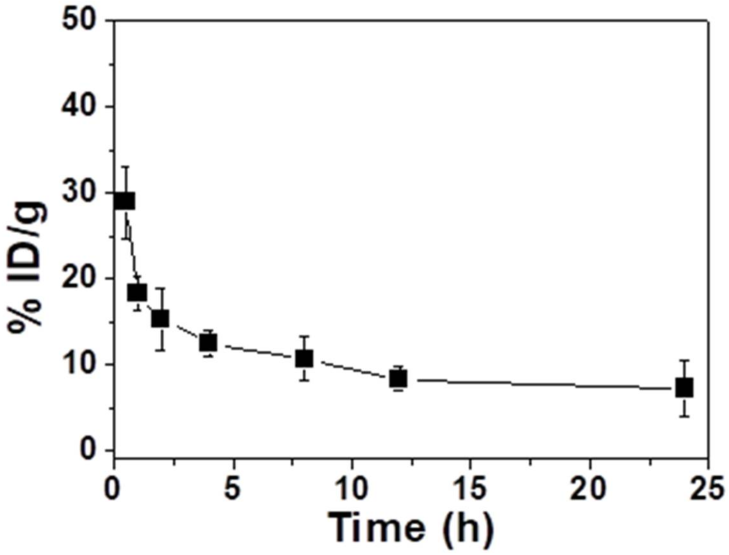

Figure S19. Blood circulation of BM-PEG-Cy7 NSs after *i.v.* injection.

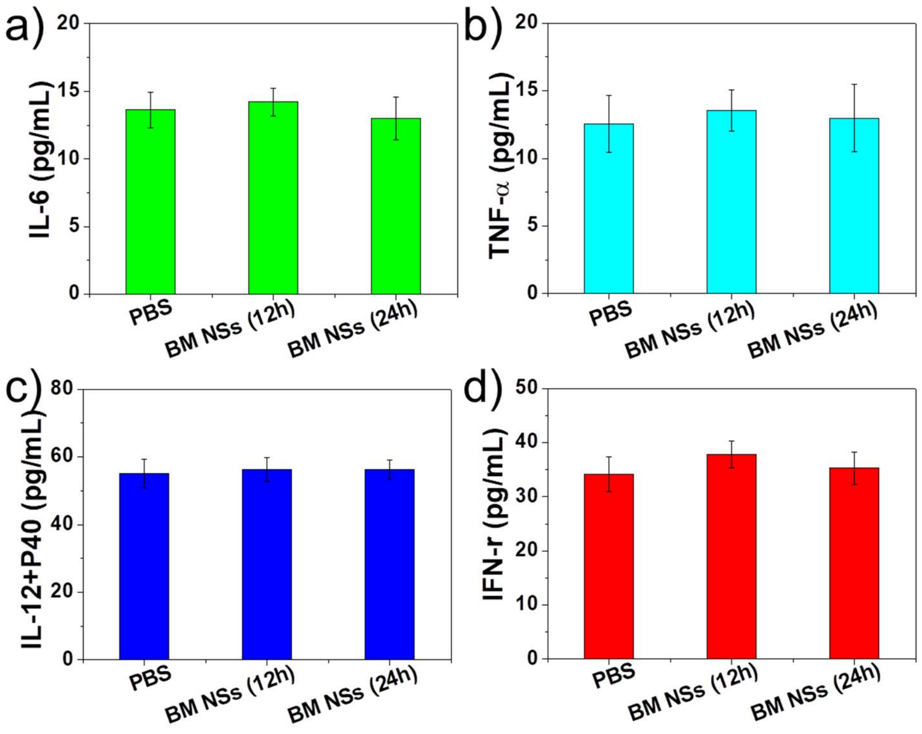

Figure S20. Serum levels of a) interleukin 6 (IL-6), b) tumor necrosis factor-α (TNF-α), c) interleukin-12 (IL-12+P40) and d) interferon-γ (IFN-γ) in healthy mice at 12 and 24 h after intravenous injection of PBS versus BM-PEG NSs.

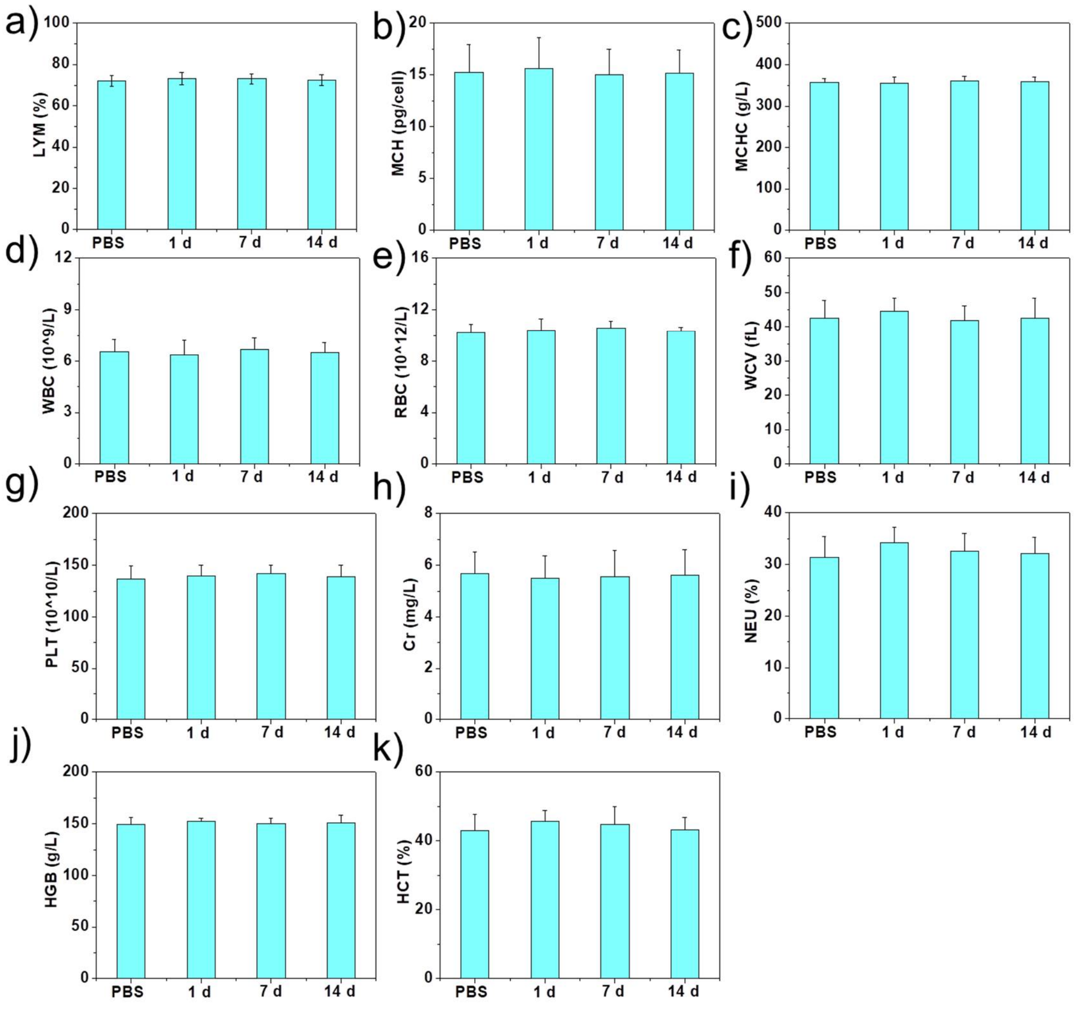

Figure S21. The detection of lymphocyte (LYM), mean corpuscular hemoglobin (MCH), mean corpuscular hemoglobin concentration (MCHC), white blood cells (WBC), red blood cells (RBC), mean corpuscular volume (MCV), platelet (PLT), creatinine (Cr), neutrophil (NEU), hemoglobin (HGB) and hematocrit (HCT) at 1, 7, and 14 d after intravenous injection of PBS versus BM-PEG NSs.

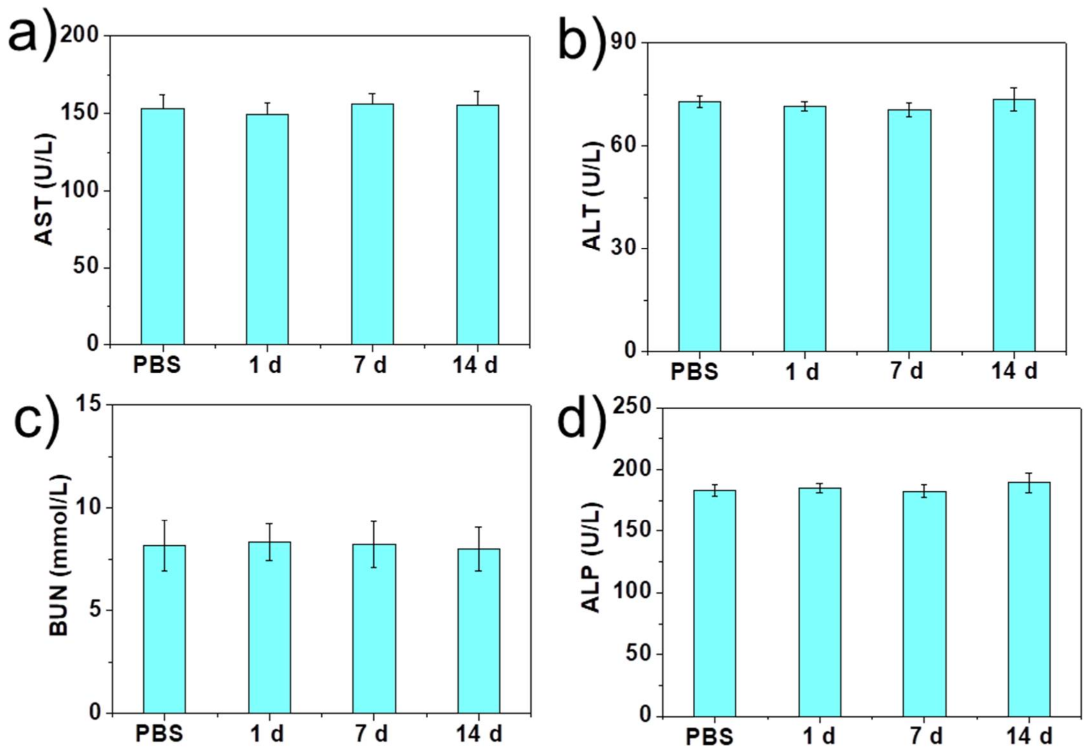

Figure S22. The detection of aspartate aminotransferase (AST), alanine aminotransferase (ALT), urea nitrogen (BUN) and alkaline phosphatase (ALP) at 1, 7, and 14 d after intravenous injection of PBS versus BM-PEG NSs.
